# Development of noncytotoxic silver–chitosan nanocomposites for efficient control of biofilm forming microbes†
†Electronic supplementary information (ESI) available: ICP-MS, DLS, FTIR, contact angle measurements, TEM/EDS, cytotoxicity results. See DOI: 10.1039/c7ra08359a


**DOI:** 10.1039/c7ra08359a

**Published:** 2017-11-13

**Authors:** Anna Regiel-Futyra, Małgorzata Kus-Liśkiewicz, Victor Sebastian, Silvia Irusta, Manuel Arruebo, Agnieszka Kyzioł, Grażyna Stochel

**Affiliations:** a Faculty of Chemistry, Jagiellonian University, Ingardena 3, 30-060 Kraków, Poland. Email: regiel@chemia.uj.edu.pl; Email: stochel@chemia.uj.edu.pl; b Faculty of Biotechnology, Biotechnology Centre for Applied and Fundamental Sciences, University of Rzeszów, Sokołowska 26, Kolbuszowa, 36-100, Poland; c Department of Chemical Engineering, Nanoscience Institute of Aragon (INA), University of Zaragoza, 50018 Zaragoza, Spain. Email: arruebom@unizar.es; d Networking Research Center on Bioengineering, Biomaterials and Nanomedicine, CIBER-BBN, 50018 Zaragoza, Spain

## Abstract

Severe bacterial and fungal infections have become a major clinical and public health concern. Nowadays, additional efforts are needed to develop effective antimicrobial materials that are not harmful to human cells. This work describes the synthesis and characterization of chitosan–ascorbic acid–silver nanocomposites as films exhibiting high antimicrobial activity and non-cytotoxicity towards human cells. The reductive and stabilizing activity of both the biocompatible polymer chitosan and ascorbic acid were used in the synthesis of silver nanoparticles (AgNPs). Herein, we propose an improved composite synthesis based on medium average molecular weight chitosan with a high deacetylation degree, that together with ascorbic acid gave films with a uniform distribution of small AgNPs (<10 nm) exhibiting high antimicrobial activity against biofilm forming bacterial and fungal strains of *Staphylococcus aureus*, *Pseudomonas aeruginosa*, *Escherichia coli* and *Candida albicans*. At the same time, the resulting solid nanocomposites showed, at the same doses, reduced or totally excluded cytotoxicity on mammalian somatic and tumoral cells. Data obtained in the present study suggest that adequately designed chitosan–silver nanocomposites are powerful and promising materials for reducing pathogenic microorganism-associated infections without harmful effects towards mammalian cells.

## Abstract 2

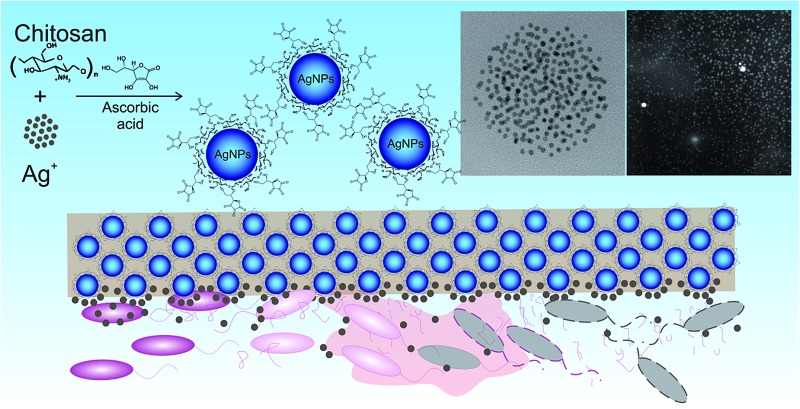
We describe the synthetic pathway to produce efficient bactericidal, fungicidal and non-cytotoxic chitosan–ascorbic acid–silver composites as solid films.

## Introduction

The increase of antibiotic-resistant bacteria and fungi is a rising concern worldwide. The extensive and routine use of first-line antimicrobials in medicine, agriculture, and farming over the last few decades has led a major threat for clinical practice. The World Health Organization (WHO) has recognized antibiotic resistance as one of the three major threats to global health and mankind.^1^ Several resistance mechanisms, such as enzymes destroying antibiotics or biofilm formation, have emerged, making some of the antimicrobials virtually ineffective. Antimicrobial resistance (AMR) threatens the effective treatment of a wide range of infections which are able to spread, imposing a huge cost for healthcare systems. Multidrug-resistant (MDR) strain-, tuberculosis- (TB) or methicillin-resistant *Staphylococcus aureus* (MRSA)-associated infections lead to several serious diseases, requiring prolonged and intensive hospital care, while the number of effective antibiotics against them is very limited. Even the new generation of antibiotics is becoming virtually ineffective and it is predicted that AMR will cause more deaths than cancer diseases by the middle of the century.^2^ We could reach a critical point where routine surgeries or minor infections could jeopardize human life and, therefore, we must act now on a global scale to slow down antimicrobial resistance.

Beside the global public awareness campaign, improving hygiene or promoting new rapid diagnostic systems to cut the unnecessary use of antibiotics, the development of effective alternative approaches in the antibacterial field is particularly relevant and urgent. Nanotechnology has become increasingly utilized in the medical field and it has been widely applied in reducing the activity of numerous resistant microorganisms. Several pioneering studies presented nanomaterials as potential antiseptics and disinfectants with novel modes of action and multiple microbial targets compared with traditional antibiotics. Among all the active substances presented in the literature, metal nanoparticles (*e.g.* silver, copper, gold) arise as potent antimicrobial agents towards Gram-positive, Gram-negative bacteria and fungal strains.^3^–^5^

The antiseptic activity of silver-based compounds against a broad spectrum of microorganisms has been known for centuries. The chemical and heat resistance of silver nanoparticles caused their wide utilization as antibacterial agents. Thanks to their tunable properties such as size, shape and progressive surface oxidation resulting in silver ions release, have gathered a lot of attention. Nowadays, several silver-based medical products have been developed to control microbial proliferation, such as topical creams, antimicrobial bandages, wound healing dressings, silver-lined endotracheal tubes or surgical meshes being efficiently implemented.^6^ Moreover, silver-based antibacterial materials found application in a wide variety of daily life products such as drinking water purification systems, textiles, home appliances and food-preservatives.^7^,^8^ When silver is present in its nanoparticulated form, it can rapidly oxidize releasing silver ions due to the large surface area per volume ratio of nanoparticles, acting the silver nanoparticle as a silver ions depot.

The exact mode of silver biocidal action is not fully understood. Several studies suggest that silver nanoparticles interact physically with the cell wall and consequently its penetration leads to cell disruption.^9^ Other studies assign the antimicrobial mode of AgNPs to the silver ions released from the NPs' surface.^10^ Silver ions can bind to sulfhydryl groups leading to protein denaturation or they can attach to the cell membrane, and thus change its permeability. Another hypothesis of the silver antimicrobial action proposes the dissipation of the proton motive force^10^ or the generation of reactive oxygen species and free radicals which mediate cell membrane damage and death.^11^ One common assumption is that the antimicrobial activity depends mostly on the size of nanoparticles. The smaller diameters, the higher the surface to volume ratio and thus the enhanced surface oxidation and consequent ions release, resulting in the increased bactericidal action when compared to the same bulk material.^12^ Nevertheless, a broad discussion concerning the side effects towards mammalian cells and the environment is still open when developing silver-based biomedical materials.^13^

One of the most promising approach in the development of novel antibacterial and efficient composites is the utilization of biocompatible polymers such as polyethylene glycol (PEG), polyvinyl alcohol (PVA), polyacrylic acid (PAA) as host matrices for the antimicrobial moiety. The incorporation of metal nanoparticles in the polymeric matrix or their direct surface modification with polymers can significantly improve the biological action towards both – microorganisms and mammalian cells and prevents from an uncontrolled release of the nanoparticles to the environment. In addition, such hybrid nanomaterials may tune the bactericidal Ag^+^ delivery with a fast initial and subsequent sustained release at the infection site. Depending on the application, different release profiles are usually required and it is highly desirable to control the active agent release from the polymeric matrix.

Among several synthetic approaches for metallic nanoparticles formation, the polymer-based route has gathered a lot of attention.^14^,^15^ Several facile routes of silver nanoparticles synthesis with chitosan as mediator have been presented in the literature.^10^ Chitosan (CS) is a polycationic aminopolysaccharide obtained by the *N*-deacetylation of chitin, the main ingredient of crustacean shells, fungi or insects. This biocompatible and biodegradable polymer thanks to its appealing properties resulting from the presence of functional groups (amino and hydroxyl) has found several biomedical applications.^14^ Beside its chelating and sorbent ability, CS is known to be non-toxic and does not induce allergic and inflammatory reactions after implantation or topical application on the human body. Moreover, chitosan displays antimicrobial activity against both Gram-positive and Gram-negative bacterial strains.^16^ Its biological activity significantly depends on the polymer properties such as molecular weight (*M*_w_) and deacetylation degree (DD). In our previous studies, we have presented an insightful study on chitosan-based silver nanoparticles synthesis with a strong emphasis laid on the influence of the polymer molecular weight and deacetylation degree on the resulting properties of AgNPs and composites and their antibacterial action.^3^

In this current work, we have continued the optimization of the synthetic pathway to produce efficient bactericidal composites as solid films. Here we present an improved synthetic procedure which renders a large amount of ultra-small AgNPs properly dispersed in the chitosan polymeric matrix thanks to the use of a second reducing agent (*i.e.*, ascorbic acid, VC). Those materials while having a low metallic silver content, they still can serve as prolonged-use antibacterial agents due to the chemisorbed silver ions release from the NPs surface and simultaneously their potential adverse effects towards human cells are limited. The antimicrobial effect of the obtained composites was evaluated against biofilm forming resistant strains of *Staphylococcus aureus* ATTC 25923, *Pseudomonas aeruginosa* ATTC 27853, *Escherichia coli* PCM 2209 and against *Candida albicans* ATCC 14053 (Robin) Berkhout at different growth phases. The potential *in vitro* cytotoxic effect was evaluated towards selected human cell lines (A549, HaCaT, and CT26).

## Experimental

### Materials

Chitosan with low (L), medium (M) and high (H) average molecular weight and varying deacetylation degree (*M*_w_ ∼ 369 ± 4; 1278 ± 8; 2520 ± 9 kDa, respectively, DD ∼ 86 ± 3; 89 ± 2; 85 ± 3%, respectively^3^), were purchased from Sigma-Aldrich. Silver nitrate (AgNO_3_ ≥ 99%), ascorbic acid (≥99%), acetic acid (≥99.7%) and sodium hydroxide (anhydrous, ≥98%) were also supplied by Sigma-Aldrich. All reagents were used as received.

### Chitosan–ascorbic acid based silver nanoparticle synthesis and composites preparation

The synthesis of chitosan-based silver nanoparticles, optimized previously utilizes three grades of chitosan varying in the average *M*_w_ and DD for the reduction and stabilization of silver nanoparticle.^3^ Based on the previously obtained physicochemical characteristics of chitosan–silver nanocomposites,^3^ herein another synthetic procedure was optimized and presented. Chitosan M solutions (1% (w/v)) were heated up to 95 °C using an oil bath. Then, silver nitrate (14, 24, 52, 104 mM) and ascorbic acid (vitamin C – VC; 1 mM) solutions were added dropwise in volume ratio (CS : AgNO_3_ : VC = 5 : 1 : 1) and so prepared mixtures were kept under heating and stirring for 12–15 h (optimized experimentally). To maintain the previously optimized total volume ratio between the polymer solution and silver ions/ascorbic acid solutions, AgNO_3_ concentrations were twice concentrated, while the volume was twice reduced (replaced with ascorbic acid volume).^3^ A system of abbreviation was used in the text (*e.g.* M7/VC where M stands for chitosan with medium *M*_w_ and 7 for 7 mM initial silver precursor concentration (in the total volume of AgNO_3_ and VC solutions)) to simplify samples identification. Nanocomposites in a form of polymeric films were prepared by the solvent evaporation method. Chitosan solution and chitosan-based silver nanoparticles colloids (25 mL) were poured into polystyrene Petri dishes (internal diameter 9 cm) and dried in an electric oven (Pol-Eko) at 60 °C until a complete solvent evaporation. In a second step, chitosan and chitosan–silver nanoparticles were neutralized with 1 wt% NaOH solution and washed with deionized water. Neutralized composites were dried and kept in the dark until further use. The previously reported synthetic procedure based solely on chitosan L/M/H^3^ was also performed, in order to obtain comparative composites for biological studies.

Further in this paper, the system of abbreviation was used to distinguish the different composites prepared. Three grades of chitosan, varying in their average molecular weight and deacetylation degree, are abbreviated from now on as L, M and H. Different silver loadings are also included in the short names of the composites (7, 12, 26, 52). Materials based on chitosan with medium molecular weight together with ascorbic acid are named as M/VC.

### Chitosan/ascorbic acid–silver nanocomposites characterization

UV-Vis spectrophotometry was used as an analytical tool to track silver nanoparticles formation during synthesis. UV-Vis measurements were carried out in a double beam UV-Vis spectrophotometer (Perkin Elmer Lambda 35), between 200 and 800 nm with 1 nm step. Silver precursor and resulting nanoparticles concentrations were determined *via* inductively coupled plasma mass spectrometry (ICP-MS; Elan 6100 Spectrometer) and inductively coupled plasma optical emission spectroscopy (ICP-OES) techniques. Transmission electron microscopy (TEM) images of polymer-based nanoparticles colloids were taken using an FEI™ Tecnai T20 Microscope operated at 200 kV. Samples were diluted in ethanol and dropped on a copper grid. The size distribution of colloidal nanoparticles was determined from the enlarged TEM micrographs, using National Instruments IMAQ Vision Builder software, counting at least 200 particles in different images. Nanoparticles morphology and distribution in the polymeric matrix were analyzed by using a FEI™ Tecnai T20 microscope and a FEI™ Tecnai G2 F30 microscope operating at 300 kV in Scanning Transmission mode, equipped with a cryo-holder to avoid damage of the samples (high-resolution STEM with HAADF detector was also carried out). Silver nanoparticles were identified by collecting energy dispersive X-ray spectra (EDS; ESI†). Nanocomposites were fixed in a resin and cut with an Ultramicrotome (Leica EM UC7) equipped with a diamond knife. Silver nanoparticles distribution across the film, the oxidation state of metals and the weight percentage of NPs in the selected composites were determined by X-ray photoelectron spectroscopic measurements (Axis Ultra DLD 150, Kratos Tech.). The nanocomposites were excited by the monochromatized Al Kα source (1486.6 eV) run at 15 kV and 10 mA. Silver species release from MAg/VC solid nanocomposites was also evaluated. Briefly, the extracts were prepared by 24 h incubation of 3 × 3 cm squares of each sample in 10 mL of MiliQ water at 37 °C. The dialysis of each lixiviate was carried out (Pur-A-Lyzer™ Maxi Dialysis Tubes with MWCO 3.5 kDa) in order to separate NPs from ions and the silver species concentration was determined by ICP-MS (Elan 6100 Spectrometer).

### Bacterial and fungal cultures

The Gram-positive strain of *Staphylococcus aureus* ATCC 25923 and two Gram-negative strains of *Pseudomonas aeruginosa* ATCC 27853 and *Escherichia coli* PCM2209 were chosen as model strains exhibiting increased resistance *via* biofilm formation for the susceptibility testing. All strains were maintained in enriched Nutrient Broth (NB, BTL) and kept at 4 °C. In the initial cultures for an antimicrobial test 5–10 μL of bacteria were inoculated into 10 mL of Tryptone Soy Broth (TSB, BTL) and incubated at 37 °C for 18–24 h to obtain ∼10^8^–10^9^ colony forming units per mL (CFU per mL). Tryptone soy agar (BTL) was used for seeding plates preparation and for the initial culture for antibacterial tests preparation (ASTM E2180-07 standard method). Phosphate buffered saline (PBS) was employed for carrying out serial dilutions. Fungal strain *Candida albicans* ATCC 14053 (Robin) Berkhout was maintained in Sabouraud Broth (SB, BTL; broth containing peptones designed for fungi cultivation) and kept at 4 °C. In the initial culture for the antimicrobial test, 10 μL of fungi were transferred and inoculated into 5 mL of SB and incubated at 28 °C for 18–24 h to obtain ∼10^9^ CFU mL^–1^. Sabouraud agar enriched with 4% of glucose (BTL) was used for seeding plates preparation and for the initial culture for antifungal tests preparation. Bacterial and fungal strains cultivation was carried out in a bacteriological incubator (Thermo Scientific, MaxQ 6000). All assays were carried out in a laminar flow hood (Thermo Scientific, MSC Advantage). All materials were sterilized before use in an autoclave (Prestige Medical, Classic) at 121 °C for 20 min.

### Antimicrobial test procedure

The antibacterial activity of solid nanocomposites in a direct contact form was performed according to a modified ASTM E2180-07 standard method (Standard Test Method for Determining the Activity of Incorporated Antimicrobial Agent(s) In Polymeric or Hydrophobic Materials).^17^ Two types of experiments were performed varying in the initial bacterial concentration: (i) with 10^5^–10^6^ CFU mL^–1^ corresponding to the bacterial exponential growth phase and (ii) with CFU mL^–1^ ≥ 10^7^ corresponding to the stationary growth phase of bacterial culture. Briefly, different volumes (optimized for each strain) of the overnight stationary phase bacterial culture in TSB were transferred to 100 mL of sterile agar slurry (40 °C). In the next step, 1 mL of the initial inoculum was pipetted on each polymeric sample, placed on a sterile Petri plate inside a sterile glass Petri plate. Samples with bacteria were co-incubated at 37 °C for 18–24 h in the dark. After incubation, polymeric samples were transferred to test tubes containing 10 mL of TSB. Tubes were treated with ultrasounds for 2 minutes in order to detach potentially survived fractions of bacteria, stirred with vortex and series of decimal dilutions for each tube were carried out in PBS. Spreading of the dilutions on agar plates was performed (3 × 20 μL) in order to obtain representative CFU values. Viable bacterial colonies were counted after 24 h of incubation and CFU mL^–1^ were calculated. Each sample and control (polymer without AgNPs) were tested in at least triplicate biological repetitions. Results were presented as mean values of log(CFU) with standard deviation. A detailed description of the procedure was previously described.^5^ Antifungal activity of MAg/VC nanocomposites against *Candida albicans* ATCC 14053 was also evaluated according to the modified ASTM E2180-07 standard test method, adapted for fungi. The experiment was performed with the initial fungal concentration ∼10^5^ CFU mL^–1^ corresponding to the exponential growth phase. The temperature of fungal cultures cultivation was decreased to 28 °C and Sabouraud broth/agar were used.

### Antimicrobial activity test without direct sample contact with bacteria

The antibacterial effectiveness was also evaluated in a non-contact form. In this approach, the extracts released from chitosan–silver films were incubated with bacterial cultures. The corresponding nanocomposites were incubated at 37 °C for 24 h in sterile test tubes containing 9 mL of PBS. Afterward, films were removed and 1 mL of bacterial culture in TSB was added to the tubes in order to obtain 10^5^–10^6^ CFU mL^–1^ and incubated at 37 °C for 24 h. Pure PBS with bacteria addition was treated as an initial control and control after 24 h. Series of decimal dilutions and spreading on agar plates was performed (3 × 20 μL). Viable bacterial colonies were counted after 24 h of incubation. Results were presented as mean values of log(CFU) with standard deviation. Each sample was tested in at least triplicate biological repetitions.

Nanocomposites, which were used for the extracts preparation were again tested in a direct contact form according to ASTM E2180-07 standard method described above. The aim of this experiment was to elucidate whether the potential release of unbound silver species from the films during extracts preparation influences their bactericidal action.

### *In vitro* cytotoxicity study

The cytotoxicity studies were performed on three selected cell lines: murine colon carcinoma CT26.WT (fibroblasts, ATCC CRL-2638™, LCG Standards, Barcelona, Spain), human lung adenocarcinoma A549 (epithelial, ATCC CCL-185™, from LCG Standards, Barcelona, Spain) and human keratinocytes HaCaT (kindly donated by Dr Olexandr Korchynskyi and Prof. Mykhailo Gonchar). Cells were routinely cultured in high-glucose (4.5 g L^–1^) Dulbecco's Modified Eagle's Medium (DMEM) with l-glutamine and phenol red (Lonza) supplemented with 10% Fetal Bovine Serum (FBS) and 1% antibiotics – streptomycin (100 μg mL^–1^), penicillin (100 U mL^–1^), Amphotericin Mix (Thermo-Scientific). Cells were cultured at 37 °C in a humidified incubator in a 5% CO_2_ atmosphere. The *in vitro* cytotoxic effect of chitosan-based composites with silver nanoparticles was assessed according to a modified, previously described method, using material extracts.^6^,^18^,^19^ Since chitosan is known to be nontoxic and biocompatible,^20^ the potential short contact cytotoxic effect might be attributed to the metallic species (nanoparticles or ions) released from the composites. The extracts of polymeric films were prepared by incubating pure polymeric films and composites with NPs (cast previously in 12-well plates and sterilized under UV light, 30 minutes) in DMEM with 1% antibiotics without FBS for 24 h at 37 °C in a humidified incubator with a 5% CO_2_ atmosphere. Each sample was used twice for the extracts preparation to assess the difference in biological activity after washing out the unbound silver species. Cells were seeded in 24-well flat-bottom plates with a density 5 × 10^4^, cultivated 24 h and afterward washed with PBS (Lonza). The effect of extracts treatment on cells was evaluated in two variants: (i) 300 μL of pure extract (without serum; S(–)) and (ii) 300 μL of extract supplemented with 300 μL of fresh medium with serum (S(+)). Cells treated with the corresponding volume of culture medium were also treated as control. Cell viability after 24 h of incubation was assessed *via* the MTT and Alamar Blue assays,^21^ (both supplied by Sigma-Aldrich; the procedure was described in detail in the ESI†). The results were presented as average values of three independent experiments with minimum 4 repetitions in each viability test ± standard deviation.

### Cells morphology studies after treatment with extracts (fluorescence microscopy)

The influence of nanocomposites treatment on HaCaT and A549 cells morphology and potential disruption of organelles were evaluated through fluorescence staining. Cells previously seeded in 12 well plates with density 1.5 × 10^5^ and 1.0 × 10^5^ cells/well, respectively were treated with chitosan–silver nanocomposites extracts (M52/VC) and co-incubated (24 h at 37 °C). Afterward, cells were washed with PBS (Ca^2+^, Mg^2+^) in triplicate and stained with organelle-specific dyes according to the manufacturer manuals.

Briefly, mitochondria were specifically stained with 100 μM solution of Mito Tracker® Green FM (Molecular Probes; Life Technologies; 470–495 nm excitation filter) in PBS with Ca^2+^, Mg^2+^. Fixation and permeabilization of cells, conducted before the F-actin visualization, were carried out by incubating the cells with 2% paraformaldehyde solution in PBS for 10 minutes at room temperature. Actin was stained with ActinGreen™ 488 ReadyProbes® Reagent (2 drops/1 mL of medium) (Molecular Probes; Life Technologies; 470–495 nm excitation filter) for 30 minutes at 37 °C. Cellular nuclei were stained with 2 μg mL^–1^ Hoechst 33258 (Sigma-Aldrich; 530–550 nm excitation filter) solution in PBS for 10–20 minutes (for fixed and unfixed cells respectively) at 37 °C. An Olympus fluorescence microscope IX51 equipped with an XC10 camera was used for their visualization.

### Cells morphology studies after treatment with extracts (scanning electron microscopy)

The damage and potential rupture in the A549 and HaCaT cell membranes during the exposure to the chitosan–silver nanocomposite extracts were visualized by SEM. Cells were seeded on poly-l-lysine-treated glass coverslips placed in a 12-well plate with 1.5 × 10^5^ and 1.0 × 10^5^ density, respectively and incubated with chitosan–silver nanocomposites extracts (M52/VC) for 24 h. Afterward, 1 mL of fixing buffer (sodium cacodylate with 2.5 wt% glutaraldehyde and 0.1 M sucrose; Sigma-Aldrich) was added and cells were incubated at 37 °C for 1.5 h. Subsequently, fixed cells were dehydrated in a gradient of methanol. Samples were then dried at 37 °C, mounted on SEM stubs, and sputtered with a 20 nm gold layer to allow SEM observation.

## Results and discussion

### Synthetic pathway and progress tracking *via* UV-Vis measurements

The application of chitosan in the synthesis of metallic nanoparticles is a perfect example of a green and environmentally friendly synthesis.^14^ The silver nanoparticle synthesis based on chitosan (CS) serving as both, reducing and stabilizing agent, developed in our previous studies,^3^ has been here utilized for a further synthetic pathway improvement. Previously, different variables, such as polymer average molecular weight and deacetylation degree, influencing AgNPs formation, have been taken into account during the synthesis design.^3^ Thanks to the presence of numerous amino and hydroxyl groups in the CS chains, silver ions undergo coordination and afterward reduction at the applied temperature of 95 °C, coupled with the oxidation of chitosan hydroxyl groups and/or its hydrolysates.^22^ The resulting nanoparticles were bound to the polymer functional groups providing them with long-term stability. The thermodynamically-driven processes of NP's agglomeration were prevented by the polymer presence. We have shown a strong dependence between the reduction progress and substrates concentration, the reaction temperature and polymer properties. Physicochemical characterization and antibacterial activity tests revealed the most promising results for composites based on chitosan with medium molecular weight. The smallest silver nanoparticle diameters and uniform dispersion in the polymeric films provided the composites with a capacity of total eradication of biofilm forming and antibiotic resistant *Staphylococcus aureus* ATCC 6538 and 9213 strains. A total bactericidal effect was obtained only for the highest silver content. Due to the low reduction rate in the case of low silver concentrations, observed *via* surface plasmon resonance, a lower amount of AgNPs and thus less pronounced antibacterial activity was demonstrated.^3^

**Fig. 1:**
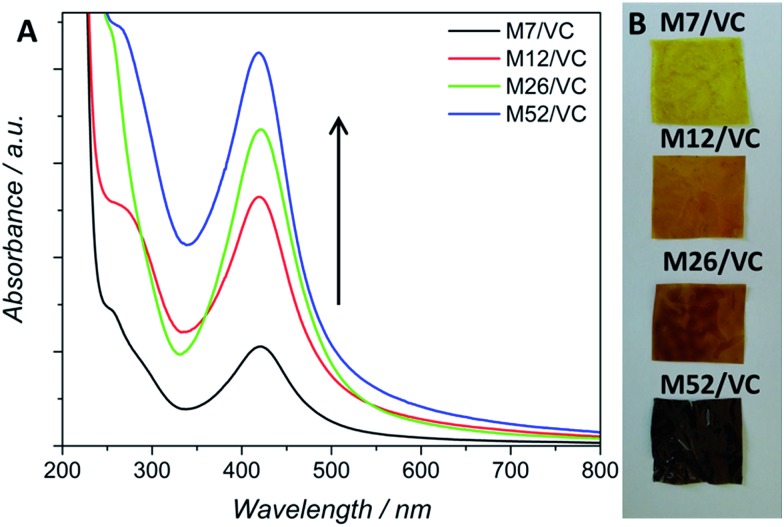
(A) UV-Vis absorption spectra for chitosan medium *M*_w_ and ascorbic acid (M/VC) based AgNPs (for 7, 12, 26 and 52 mM silver precursor). Surface plasmon resonance band (SPR) at ∼420 nm appears due to the common excitation of the nanoparticle free electrons. Black arrow depicts the increasing concentration of AgNPs. (B) Photographs of chitosan–silver films with different AgNP loadings (7, 12, 26 and 52 mM Ag^+^ initial concentration, respectively).

Based on those previous results the aim of the here presented synthetic procedure was to improve the *in situ* silver ions reduction rate even at low concentrations in order to obtain a larger amount of small AgNPs properly dispersed in the polymeric matrix. The presence of small NPs instead of silver ions could provide a long-term antimicrobial activity, resulting from the progressive surface oxidation and chemisorbed ions release while simultaneously the potential adverse effects towards human cells could be limited. Taking into account the potential toxicity and environmental requirements, only mild reducing agents were considered for the synthesis optimization *e.g.* ascorbic acid (VC). VC is a well-known reductant in the synthesis of metallic nanoparticles.^23^ Typical surface plasmon resonance band (SPR) of colloidal silver nanoparticles, resulting from the common excitation of free conducting electrons upon the interaction with the incident light, appears in the range of 350–600 nm,^24^ depending on different factors such as NP's size and shape. The UV-Vis spectra, with a characteristic SPR absorption band at ∼420 nm were collected after 12 h of synthesis (Fig. 1A). As expected, with the increase of silver ions concentration in the synthesis, a higher intensity of the SPR band was observed, confirming the reduction progress. Moreover, the characteristic SPR peak appears even at low silver nitrate concentrations in contrast to our previously obtained results without the VC addition. A narrow absorption band and much higher intensity after 12 h, when compared to the previous synthetic route, indicated a higher reduction rate due to the presence of the additional reducing agent and proved a spherical shape of the resulting nanoparticles. Herein, silver ions undergo reduction by both, chitosan functional groups and ascorbic acid.^25^ Availability of a large number of amino groups (high DD) and hydroxyl groups of vitamin C (VC) decreased the time of reduction and growth thanks to a large number of nucleation seeds and thus high silver reduction rate even at low Ag^+^ concentrations. Moreover, the potential nanoparticles migration and agglomeration were prevented due to an adequate polymer viscosity. As demonstrated in our previous report,^3^ too high *M*_w_ and thus the viscosity of the polymer solution, not only prevents the NPs coalescence but also limits the diffusion of reactants to growing nuclei leading in consequence to fewer nucleation events. Fig. 1B shows nanocomposites as films, obtained *via* the solvent evaporation technique with increasing silver loadings. The higher silver loading the darker the color of the resulting nanocomposite.

### Studies of nanoparticles morphology (size, shape) and distribution in the polymer *via* transmission electron microscopy

**Fig. 2:**
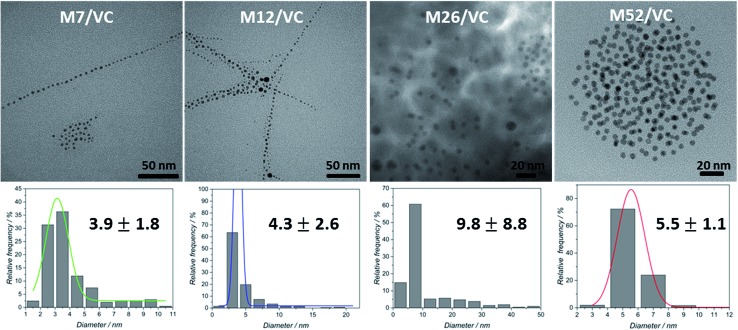
TEM photographs for MAg/VC (colloids) based on different silver precursor concentrations used in the synthesis (7, 12, 26 and 52 mM) and corresponding statistical analysis of the NP sizes based on the obtained micrographs.

**Fig. 3:**
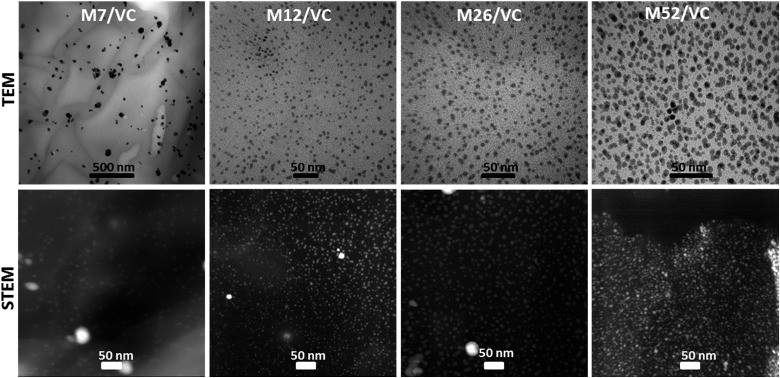
TEM (top row) and STEM-HAADF (bottom row) images of chitosan medium and ascorbic acid-based films MAg/VC with increasing silver content. A uniform distribution of nanoparticles across the film is visible. The presence of large agglomerates is excluded even with the highest silver loading.

TEM micrographs of the MAg/VC (7, 12, 26, 52 mM silver precursor) colloids revealed the formation of mainly spherical silver particles (Fig. 2). Statistical analysis (the National Instruments IMAQ Vision Builder software; *N*_min_ = 200 particles) of the NP sizes based on the obtained micrographs for different silver initial concentration confirms the presence of mostly small nanoparticles with a diameter below 10 nm directly related to the atomic number. A uniform distribution of small AgNP was observed for all composites varying in the silver content (Fig. 3). This proves a high stabilizing potential of chitosan with medium molecular weight as it has been previously established.^3^ Chitosan with medium *M*_w_ in pair with TEM images of solid composites recorded in a bright and dark field technique gave an insight into the uniformity of the NP distribution among the films. STEM-HAADF images present silver nanoparticles as bright dots because the contrast is ascorbic acid represents a compromise scenario, where a fast reduction and adequate viscosity result in small NPs formation and their uniform distribution in the polymeric film.

### X-ray photoelectron spectroscopy analysis of MAg/VC composites surface

**Fig. 4:**
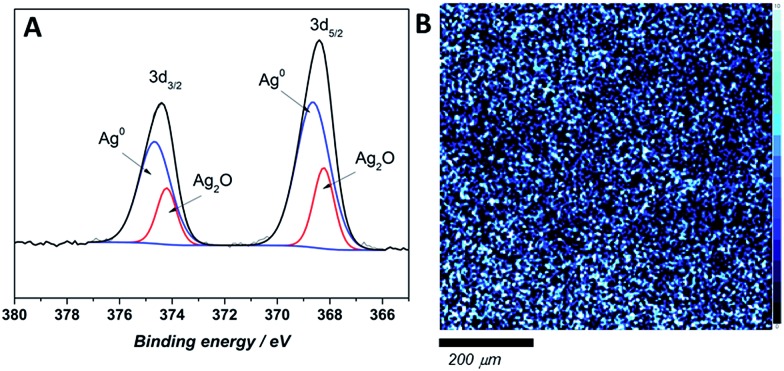
High-resolution chemical state XPS spectra of aged M26/VC composite (A). XPS map depicting silver nanoparticles distribution across the few atomic layers of M26/VC film (B). The lighter the blue, the higher the total silver content.

The chemical state and surface content of silver species in the obtained films were confirmed by X-ray photoelectron spectroscopy (XPS). In the recorded spectra of all tested composites two peaks at ∼374–375 and 367–368 eV, corresponding to Ag 3d_3/2_ and Ag 3d_5/2_ respectively, are clearly visible (Fig. 4). Scans were collected for composites prepared directly before the measurement (fresh samples) and for materials after a few weeks (aged samples). The silver 3d_5/2_ band positions for fresh composites with different silver content confirmed the presence of solely metallic silver on the uppermost atomic layers (Table 1). Also, the band shape on high-resolution scan did not reveal the presence of ionic silver. On the contrary, the high-resolution XPS scans of aged samples revealed that both of peaks, Ag 3d_3/2_, and Ag 3d_5/2_, split into two other peaks. Ag 3d_3/2_ splits into ∼374 and ∼374.7 eV, which might be attributed to the presence of ionic and metallic silver in sample.^26^,^27^ Similarly, the Ag 3d_5/2_ also splits into ∼368.7 and 367.9 eV binding energies, which confirms the previous assumption of Ag^+^ and Ag^0^ presence.^28^,^29^ Since no Ag^+^ was detected on the surface of fresh samples, ionic silver occurrence might be an effect of NP's surface oxidation proceeding over time upon contact with air. Ag 3d_5/2_ XPS map of the composite surface shows a homogenous silver distribution in samples across 10 nm depth (Fig. 4B).

**Table 1:** Binding energies of 3d_5/2_ silver species

Sample	Binding energy (3d_5/2_)/eV			
	Fresh samples		Aged ∼5 weeks	
	Ag^0^	Ag_2_O	Ag^0^	Ag_2_O
M7/VC	368.9	—	368.8	368.1
	100%		62%	38%
M12/VC	368.7	—	368.7	368.0
	100%		43%	57%
M26/VC	368.8	—	368.8	367.8
	100%		35%	65%
M52/VC	368.7	—	369.0	368.0
	100%		30%	70%

The silver species quantification based on the integrated 3d band for fresh and aged composites is listed in Table 1. As the overall silver content increases, the ionic silver contribution on the surface of the composite becomes more prominent for the aged composites. The XPS photoelectric peaks positions with atomic% of detected elements (carbon, oxygen, nitrogen and silver) were listed in Table S3 in (ESI†). The weight percentage of silver content was approximated to the theoretical ones with a difference in the 1% range.

### Silver species release from MAg/VC composites

The potential detachment of the embedded silver species was evaluated after subjecting the films to simple immersion in water for 24 h. Table 2 presents the content in silver species released from the chitosan medium and vitamin C based composites, assessed *via* ICP-MS measurements. The distinction between released ions and nanoparticles was carried out through the use of a dialysis separation of the extracts obtained from each composite in triplicate (Pur-A-Lyzer™ Maxi Dialysis Tubes with MWCO 3.5 kDa). Samples before and after separation were analyzed and the results revealed that almost 99% of released silver was ionic.

**Table 2:** Concentrations of silver species released from MAg/VC composites

Material	Total released silver concentration^*a*^/μM	Silver concentration after dialysis^*b*^/μM	% of ionic silver
M7/VC	797.7 ± 134.2	4.7 ± 3.2	99.41
M12/VC	1372.1 ± 288.6	9.2 ± 6.2	99.33
M26/VC	1375.5 ± 85.2	3.4 ± 1.0	99.75
M52/VC	1360.4 ± 248.5	2.9 ± 1.4	99.79

^*a*^Silver nanoparticles and silver ions.

^*b*^Silver nanoparticles.

Importantly, from the point of view of safety and potential uncontrolled side effects, the composites released a relatively small amount of nanoparticulated silver (aprox. <10 μM). Moreover, even though composites released a moderate amount of different silver species upon total immersion, the bactericidal action of films could not be exclusively addressed to this phenomenon, as it is described in the next paragraphs.

### Antibacterial and antifungal activity

**Fig. 5:**
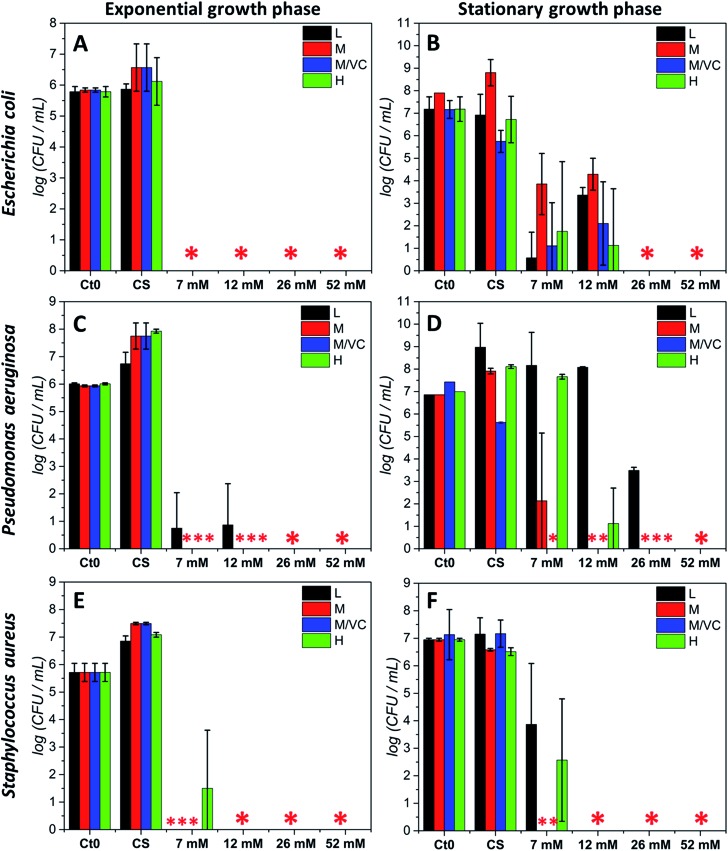
Antibacterial test results (standard norm ASTM E2180-07) for composites based on chitosan L, M, H and M/VC with different AgNPs loadings (7, 12, 26, 52 mM silver precursor), against *Escherichia coli* PCM 2209 (A, B); *Pseudomonas aeruginosa* ATCC 27853 (C, D); *Staphylococcus aureus* ATCC 25923 (E, F) in logarithmic growth phase at initial CFU mL^–1^ ∼ 10^5^ (left column) and at initial CFU mL^–1^ ∼ 10^7^ corresponding to the stationary growth phase (right column). Data were expressed as the mean ± standard error (*n* = 3). (* stands for total bactericidal effect; Ct_0_ represents the initial bacterial concentration).

The antibacterial potential of chitosan–silver composites, described in our previous paper,^3^ and chitosan/ascorbic acid silver films optimized herein, was evaluated against three representative bacterial strains according to the Standard Norm ASTM E2180-07 for polymeric substances. Two of them, *Staphylococcus aureus* and *Pseudomonas aeruginosa*, which typically populate human skin or mucous membranes and are a cause of several serious diseases.^30^,^31^ The third strain, *Escherichia coli* is a naturally occurring human colon bacterial strain, but under certain conditions can exhibit a pathogenic effect in gastrointestinal and urinary tracts. The aim of this study was to assess the antimicrobial potential of the prepared composites in two growth phases against Gram-positive and Gram-negative bacterial strains. Two variants of the initial bacterial concentration: exponential growth phase (∼10^5^ CFU mL^–1^) and stationary phase (∼10^7^ CFU mL^–1^) were utilized. The exponential phase of microorganisms growth is a pattern of balanced growth when cells are regularly dividing, growing and exhibiting an intense metabolic activity. DNA replication occurs by binary fission at a constant rate and the number of bacterial cells increases logarithmically. When the nutrients are used up by rapidly multiplying cells, the culture proceeds to the stationary phase where the reproduction rate slows down and the colony becomes stabilized. During the growth transition from logarithmic to stationary growth phase, numerous morphological and physiological changes occur such as cell shape change, volume increase, modifications in cell wall, which significantly affect their susceptibility to antibiotics and other toxic agents.^32^,^33^ Resistance increases progressively during the exponential phase, to become the strongest at the stationary phase.^33^ The antimicrobial resistance to commonly used antimicrobial agents is also dramatically increased when bacteria are able to form biofilms which exhibit a high tolerance when compared to logarithmic-phase planktonic cells.^34^ Since the efficiency of biocidal activity of chitosan–silver films against the selected biofilm forming bacteria may vary according to the growth phase, both of the presented cases were studied experimentally. Fig. 5 presents the results of the antibacterial activity against all strains in both growth phases. A total bactericidal effect was obtained for all types of nanocomposites against Gram-negative *E. coli* only in a logarithmic growth phase (Fig. 5A). In the stationary growth phase no statistically significant differences in activity towards *E. coli* between different nanocomposites were observed (Fig. 5B). For the two lower concentrations of silver (7 and 12 mM of the silver precursor) tested a reduction from 3-logs up to 6-logs in the bacterial count was obtained, while a total bactericidal effect occurred for higher silver contents (26 and 52 mM of the silver precursor). Results stay in agreement with a prediction of a superior resistance of bacterial culture in the stationary phase.

Similarly to *E. coli*, for the second Gram-negative strain *P. aeruginosa* almost a total bacterial eradication was achieved when challenging the films against bacteria in the exponential growth phase. Only 1 log of CFU mL^–1^ remained after treatment with the two lower silver contents when using chitosan L films (L7 and L12; Fig. 5C). The stationary phase of *P. aeruginosa* biofilm was more resistant to all chitosan–silver composites (Fig. 5D). M/VC composites appeared to be the most effective, and at each silver nanoparticles content level, a bactericidal effect was observed. Also, composites based solely on chitosan with medium *M*_w_ were very effective. In the case of composites based on chitosan L and H, a silver concentration-dependent activity was demonstrated. Only at the highest Ag content, total bacteria eradication was obtained for all of the tested nanocomposites. The third tested strain, Gram-positive *S. aureus* appeared to be the most susceptible for all chitosan–silver nanocomposites in both, the exponential and stationary growth phases. The bactericidal effect was achieved against *S. aureus* biofilm in both tested phases for all M and M/VC composites (Fig. 5E and F).

**Fig. 6:**
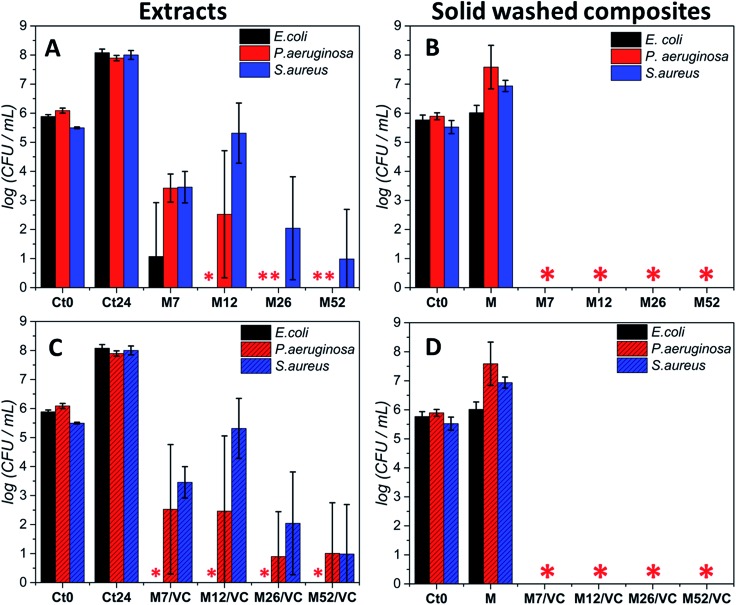
Antibacterial effect (A and C; non-contact approach) of the extracts obtained from the composites with different concentrations of silver (7, 12, 26 and 52 mM silver precursor) after co-incubation with PBS during 24 h on *Escherichia coli* PCM 2209, *Pseudomonas aeruginosa* ATCC 27853 and *Staphylococcus aureus* ATCC 25923 in logarithmic growth phase at initial CFU mL^–1^ ∼ 10^5^ (A and C). The antibacterial activity of washed composites based on chitosan M and M/VC with different AgNPs loading (7, 12, 26, 52 mM silver precursor) was also evaluated according to the (standard norm ASTM E2180-07) against the same bacterial strains in the logarithmic growth phase at initial CFU mL^–1^ ∼ 10^5^ (B and D). Data were expressed as the mean ± standard error (*n* = 3). (* stands for total bactericidal effect; Ct_0_ represents the initial bacterial concentration).

Moreover, due to the best bactericidal efficacy of M and M/VC based composites in a form of films, the antibacterial effectiveness was also evaluated in a non-contact form by co-incubating the bacterial culture in the exponential growth phase with composite extracts (Fig. 6). A concentration-dependent culture growth inhibition and reduction were observed for the tested extracts. With the increase in AgNPs concentration, a higher silver species release rate occurs and thus an elevated antibacterial effect against the selected bacterial strains was demonstrated. Interestingly, *Escherichia coli* was the most susceptible for the extracts, where even the M7/VC induced a total bactericidal effect. In contrast, *Staphylococcus aureus* appeared to be the most resistant to the extracts activity. A maximum 5 log reduction but no total bactericidal effect was observed upon 24 h incubation with the extracts.

Results stay in agreement with our previous^3^ data where a silver concentration dependent bacterial growth reduction was observed against *Staphylococcus aureus* ATCC 6538 and 9213. In both cases, extracts demonstrated a reduced activity when compared to the solid composites. Results confirm the importance of a direct contact of nanocomposites with bacteria for achieving a complete bactericidal effect. The durability of the composites antibacterial activity after the extracts preparation was assessed in the last type of test. Films used for the extracts preparation were tested in a new antibacterial experiment according to the standard norm ASTM E2180-07. Significantly, a total bactericidal effect was again confirmed for MAg and MAg/VC nanocomposites (Fig. 6B and D). The maintenance of antibacterial activity evidences a strong interaction between the chitosan amino groups and silver nanoparticles surface that enables for sustained silver ions release and thus a high performance.

The antifungal activity of chitosan–silver nanocomposites based on chitosan with medium molecular weight with and without ascorbic acid (MAg and MAg/VC; at each concentration level described previously) was evaluated against *Candida albicans* ATCC 14053 in the exponential growth phase according to modified ASTM E2180-07 standard test method, adapted for fungi. Both types of composites induced a total fungicidal effect (data not shown), which proves a high potential of chitosan–silver films in antifungal therapy. Our results stay in agreement with other studies presented in the literature, where the high silver antifungal activity was demonstrated towards several fungal strains.^35^–^39^ It is suggested that the negatively charged cell wall of *Candida albicans* undergoes a rapid interaction with AgNPs and Ag^+^ what induce the disruptions in the cell membrane leading to cell death.^36^ Moreover, Hwang *et al.* demonstrated that the elevated hydroxyl radicals production in *Candida albicans* cells upon contact with AgNPs led to mitochondrial dysfunctional apoptosis.^37^ Also, Rodríguez-Argüelles *et al.* proposed a ROS-related mechanism of antifungal and antibacterial action of chitosan–silver composites.^38^,^39^

Several composites based on chitosan and nanosilver with antimicrobial activity have been developed in the past decade as an alternative solution for the bacterial and fungal infections.^6^,^12^,^25^,^35^,^38^,^40^–^43^ Wei *et al.* demonstrated chitosan–silver nanoparticles films with high antimicrobial potential against both Gram-(+) and Gram-(–) bacteria strains. A dual activity mode was proposed, resulting from the antibacterial effect of cationic chitosan and high bactericidal action of silver.^14^ The biocidal effect of silver was attributed to the activity of both, silver ions released and chemisorbed on the NPs surface. Also, Travan *et al.* developed a biocidal chitosan-derived polysaccharide (Chitlac) based silver nanoparticle–hydrogel.^6^ A cell membrane depolarization and remarkable permeabilizing effect suggesting huge cell membrane damage induced by Chitlac-Ag when compared to the control were identified as responsible for the antibacterial action. The extracts received from Chitlac-Ag-alginate hydrogels appeared to be non-toxic towards mouse fibroblasts (NIH-3T3), human hepatocarcinoma cells (HepG2), and human osteosarcoma (MG63) cells. The exact mechanisms of both, chitosan and silver nanoparticles antibacterial activity have not been fully elucidated yet.^16^,^44^–^46^ It is claimed that the antimicrobial nature of chitosan results from the interactions between its protonated amino groups and negatively charged cell wall of microorganisms which lead to the intracellular components leakage.^47^ Both, nanoparticles and silver ions released from the nanoparticles surface are responsible for the interactions with the cell membrane, its penetration, leakage, dissipation of proton motive force, protein denaturation or ROS generation and in consequence cell death.^9^–^11^

Herein, the best antimicrobial activity was achieved for materials based on chitosan with medium molecular weight both, with and without ascorbic acid (MAg and MAg/VC). Chitosan with medium *M*_w_ enabled a uniform distribution of small, mostly spherical, nanoparticles in the polymeric film, showing enhanced surface to mass ratio prone to oxidation and thus a high concentration of chemisorbed Ag^+^ on the surface of NPs. The direct contact of pathogen cells with the composites ensures the transfer of chemisorbed ions. In contrast to the synthesis based solely on chitosan M, the addition of ascorbic acid produced well-defined spherical silver nanoparticles even at the lowest concentrations of precursor. In our experiments, we have shown that besides the silver NPs concentration both, the chitosan properties (*M*_w_, DD), and external factors (bacterial strain and initial CFU mL^–1^) can significantly influence the extent of the antibacterial effect. Direct contact between pathogenic cells and composite is necessary to achieve a total biocidal effect even at the lowest silver content (*e.g.* M7 and M7/VC). Interestingly, the extracts did not induce total bacterial cells inhibition, while the MAg and MAg/VC composites remain bactericidal against three selected bacterial strains even after the extracts collection. The XPS measurements together with silver species release study confirmed the strong entrapment of silver nanoparticles in the polymeric matrix and the presence of ionic silver on the top of the surface of composites, resulting from a progressive oxidation. Importantly, though silver ions release to the aqueous media upon immersion, the long-lasting antimicrobial effectiveness was proven. It is claimed that the antibacterial activity of silver occurs only when the silver oxide top layer is present.^48^ Importantly, the antibacterial effect of chitosan–silver composites was achieved at both exponential, and stationary growth phases. The superior resistance of biofilm forming strains to biocidal agents in stationary phase found a confirmation in the presented results. An elevated silver content was necessary to obtain a total bactericidal effect in the stationary phase when compared to logarithmic phase. Herein, the antimicrobial agent, silver nanoparticles, incorporated in the polymeric surface might induce a biocidal effect, and thus effectively prevent the bacterial adhesion and biofilm formation before getting established.

### Cytotoxicity studies

**Fig. 7:**
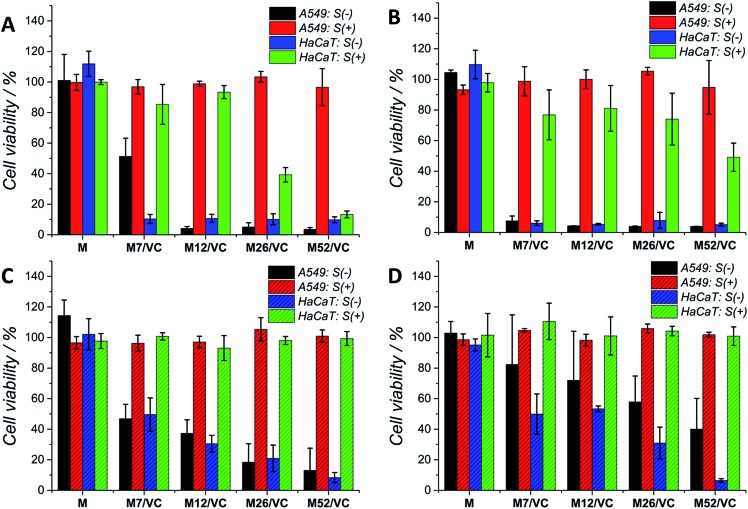
The cytotoxic effect of chitosan medium and ascorbic acid based composites – MAg/VC 1^st^ (A and B) and 2^nd^ extracts (C and D) on A549 and HaCaT cell lines after incubation for 24 h. Cell viability was assessed by the Alamar Blue (left column) and MTT (right column) test with (S(+)) and without (S(–)) serum proteins.

Although silver nanoparticles exhibit a huge antimicrobial activity, the side effects towards human cells are limiting their therapeutic applications.^13^,^49^–^52^ They are known to induce cytotoxicity and genotoxicity or oxidation stress and inflammatory response. Therefore, the potential application of composites containing metal nanoparticles in the medical field demands their biosafety assessment. Herein, the cytotoxicity of chitosan–silver nanocomposites was studied on three selected cell lines: human lung adenocarcinoma cells A549, human keratinocyte HaCaT and murine colon carcinoma CT26 (ESI†). The Alamar Blue (Fig. 7A and C) and MTT (Fig. 7B and D) tests were used to evaluate the cell viability after 24 h contact with extracts retrieved from nanocomposites. Cells were treated with 1^st^ (Fig. 7A and B) and 2^nd^ extracts (Fig. 7C and D) without serum proteins (S(–)) or extracts supplemented with fresh medium with serum proteins (S(+)). The first extracts of MAg and MAg/VC samples at each silver content without serum proteins (S–) induced almost 100% of mortality within 24 h of incubation towards A549 cell line. However, when incubated with serum proteins, almost no cytotoxic effects were observed. The cell viability in all cases was above 80%, which generally might be considered as noncytotoxic. Similarly, when cells incubated with the 2^nd^ extracts, higher toxicity was observed for samples S(–), while extracts supplemented with FBS were not toxic. The cytotoxic effect of chitosan–silver nanocomposites was also assessed on human keratinocytes, which are especially relevant in terms of the potential medical application, as for instance an antibacterial element in wound dressings. As in the case of A549 cells, a severe cytotoxicity for the 1^st^ extracts without FBS was observed for all of the tested samples. No toxic effect was demonstrated for the FBS supplemented extracts at the two lower silver contents (7 and 12 mM), whereas the cell viability was significantly reduced for the higher concentrations (26 and 52 mM). This cytotoxic effect was excluded in the case of the 2^nd^ extracts with serum proteins. Interestingly, the second extracts without serum proteins induced lower cytotoxicity, when compared to the 1^st^ extracts. The concentration dependent toxicity was demonstrated for both types of materials. This fact might be explained by the partial detachment of silver ions present on the outer layer of the composites upon the 1^st^ extracts preparation, and thus reduced the amount of Ag species upon second extracts preparation. Importantly, materials after the extract preparation remain antibacterial, while no toxicity upon incubation with serum proteins was obtained. A good correspondence between the Alamar Blue and the MTT results was obtained for both of the tested cell lines. This significant difference in the cytotoxicity of S(–) and S(+) experimental conditions might be explained by the silver neutralization with serum proteins. Silver binds to numerous amino groups of proteins, which in turns due to the reductive properties are able to reduce toxic silver ions.^53^ The mitigated cytotoxicity of nanoparticles with protein corona has been recently reported.^54^,^55^ Our results stay in agreement with Miclaus *et al.*, who demonstrated the reduction of silver NPs cytotoxicity by modulating the protein corona composition.^56^ The formation of an Ag_2_S layer on AgNPs surface upon incubation in cell culture medium with different amounts of FBS and thus protein-induced mechanism of Ag^+^ removal was demonstrated. As a consequence, the bioavailability decreases and prevention of cell death occurs. Modification of NP's surface with biomolecules significantly modulates the particle-cell interactions, however, the correlation of the protein corona composition to cellular uptake and cytotoxicity is still relatively weakly understood.^57^,^58^ Herein, the presence of oxidized silver on the top surface of chitosan–silver nanocomposites was revealed by the XPS measurements. Upon extracts preparation, silver ions are being released to the culture medium. The addition of FBS proteins reduced the cytotoxic effect probably *via* the proposed above mechanism. Importantly, results obtained in the experimental system containing FBS proteins, similar to the physiological conditions, provide and evidence for the biosafety of chitosan–silver nanocomposites in short time contacts. Results support the huge potential of those chitosan–silver nanocomposites in the biomedical field. All of the cytotoxicity results obtained for chitosan L/M/H based nanocomposites were presented in the ESI.†

### Cells morphology visualization *via* fluorescence microscopy

**Fig. 8:**
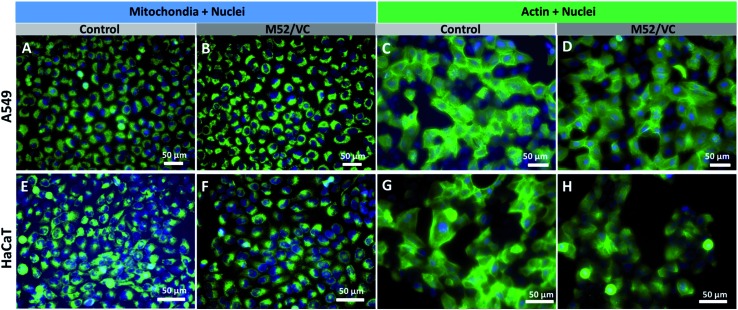
Fluorescence imaging of A549 (A–D) and HaCaT (E–H) cells after 24 h incubation with M52/VC 1^st^ extract in medium with serum proteins. Mito Tracker Green was used for mitochondria visualizing, Hoechst 33258 was used to image nuclei, while Actin Green was used for staining actin proteins was used. The green and blue colors come from the organelle-specific dyes respectively. Scale bar is 50 μm.

The actin structure of A549 cells and HaCaT was investigated to further determine whether the MAg/VC extracts induce changes in the morphology and adhesion properties of cells caused by the cytoskeleton disorder upon incubation with a serum-supplemented medium. The cellular stress induced by the activity of silver nanoparticles may manifest as disruption of the cytoskeleton network, which is critical for several cellular processes, including cell adhesion, migration or division. The oxidative stress associated with NPs internalization may also affect mitochondrial viability which can also lead to cell death.^59^ ActinGreen 488 ReadyProbes and Mito Tracker Green (Life Technologies) were used to stain the cytoskeleton and mitochondria respectively. In both cases, a co-staining of nuclei with Hoechst 33258 was performed (Fig. 8). The fluorescence micrographs of A549 cells revealed no significant changes in mitochondrial shape and size and no apoptotic bodies after incubation with M52/VC extracts with proteins (Fig. 8A and B). Moreover, extracts did not cause the actin depolarization or membrane retraction and thus no F-actin fiber disorder was proven when compared to the control sample (Fig. 8C and D). Similarly to A549 cell line, the morphology of HaCaT cells mitochondria and nuclei was not altered by M52/VC 1^st^ extract in FBS supplemented medium (Fig. 8E and F). Still, the number of mitochondria was slightly decreased after incubation with silver extract which might be a symptom of altered cell conditions. Also, no significant changes in the actin depolarization or membrane retraction were observed for HaCaT cells after incubation with M52/VC sample (Fig. 8G and H). Results are consistent with the cytotoxicity tests, where no cell viability for A549 cells and reduced cell viability for HaCaT cells were demonstrated.

### Cells morphology visualization *via* scanning electron microscopy

**Fig. 9:**
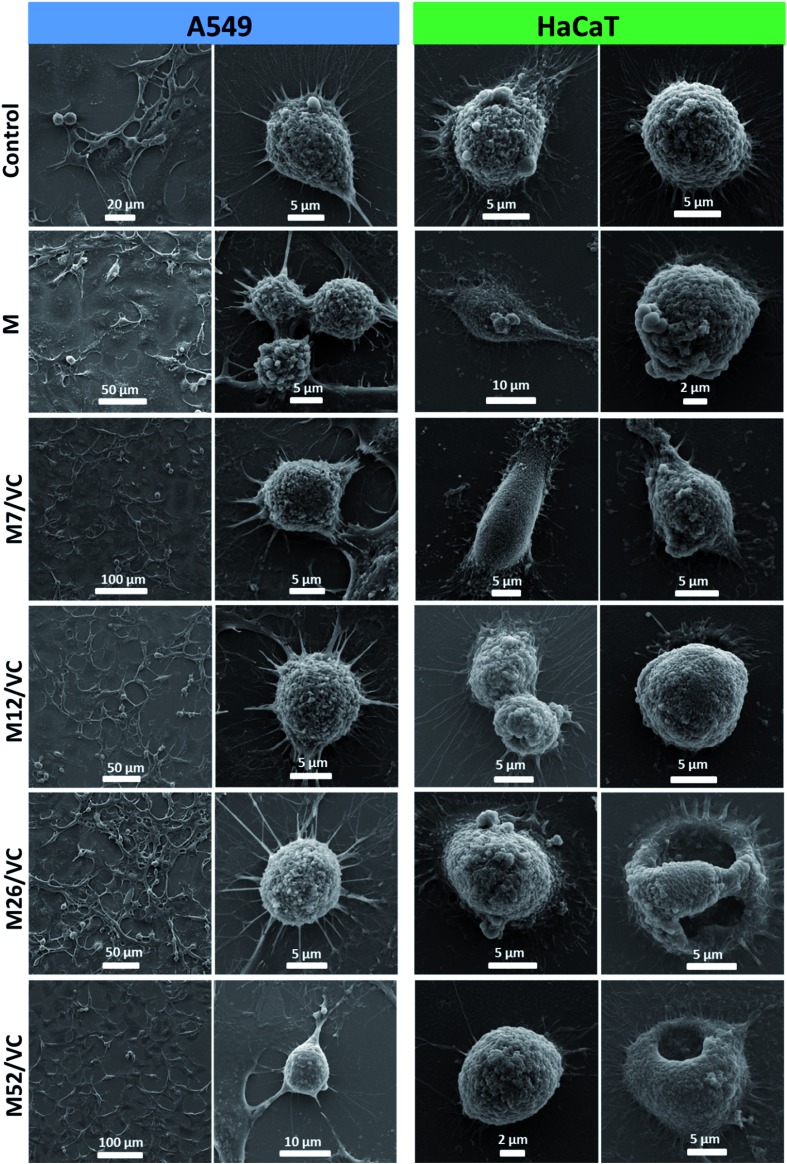
A549 and HaCaT cells morphology visualization after incubation with chitosan–silver nanocomposites 1^st^ extracts (MAg/VC) with 10% FBS.

The cell morphology was further visualized by scanning electron microscopy. Fig. 9 presents the micrographs of A549 and HaCaT cells after incubation with MAg/VC 1^st^ extracts supplemented with FBS proteins. In both cases, control sample and cells treated with pure chitosan extracts were analyzed for comparison. The A549 cell membrane integrity and morphology were not altered when compared to the control, which is consistent with the cytotoxicity tests, where no reduction in A549 cell viability was demonstrated for the tested samples. On the contrary, for human keratinocytes, silver concentration dependent morphological changes of the cell membrane were observed. Cells co-cultured with extracts possessed fewer filopodia when compared to control that might be associated with a weaker adherence to the support induced by silver. Consistently with the cell viability experiments, significant morphological changes in the cell membrane were noticed for the two higher silver contents. Among several intact and viable upon fixation HaCaT cells, a lot of disrupted cells were visualized (Fig. 9). The form of chitosan–silver nanocomposites significantly determines their biological activity. Herein, composites in the form of films were developed, optimized and tested experimentally towards bacterial, fungal and eukaryotic cells. Colloidal samples exhibit a huge antimicrobial potential due to the freely diffusing silver species and positive charge of chitosan chains, however, also the cytotoxic effect towards human cells is pronounced.^44^,^49^,^50^,^60^ When the solid polymeric matrix entraps silver nanoparticles, as in the case of *e.g.* Chitlac-based 3D structures or our chitosan films, the excessive availability of AgNPs for eukaryotic cells is prevented while the antimicrobial activity in a direct contact with pathogens surface is preserved.^6^ In this regard, the form of film possesses a huge advantage over colloids. In summary, these results point out towards the medical application potential of chitosan–silver films resulting from their considerable antimicrobial activity and simultaneous limitation of silver species release to the surrounding environment. This finding advantageously contributes to the improvement of biopolymer–silver composites safety assessment and shed light on future secure applications.

Presented herein a novel and reliable synthetic route for the preparation of chitosan–ascorbic acid–silver based nanocomposites, in which a high rate of silver ions reduction is carried out by both functional groups of chitosan and ascorbic acid. A proper stabilization of the obtained small nanoparticles within the polymeric matrix resulted in their uniform distribution in the final films. Finely tuned synthesis parameters together with specific chitosan properties determined the desirable properties for the resulting materials. A reduced size, uniform nanoparticles distribution and chemical bonds between the NPs and the polymer functional groups, confirmed through several techniques such as TEM, FTIR, XPS and silver species release study, influenced the biological activity of the composites. The antimicrobial mechanism of the composites was attributed to the direct interaction between the material surface and the microbial cells. Moreover, composites were efficient against bacterial strains in both growth phases, however, an elevated silver content was necessary to obtain a total bactericidal effect in the stationary phase when compared to the exponential phase. The cytotoxicity tests performed with the composites revealed a negligible adverse effect towards human cells when incubated with FBS proteins present in physiological conditions. These results evidence the biosafety of chitosan–silver nanocomposites in short contact times with human cells.

## Conclusions

In this work, we present the optimized pathway for chitosan–ascorbic acid–silver nanocomposites synthesis, with chitosan and ascorbic acid serving as both, reducing and stabilizing agents. The appearance of the narrow UV-Vis absorption band confirmed that the application of the second reducing agent improved the reduction rate, reduced the size (<10 nm) and provided the monodispersity even at the lowest silver content used as precursor tested when compared to the synthetic procedure based solely on chitosan. The best results in terms of, both physicochemical and biological properties, were obtained for chitosan medium and ascorbic acid based silver nanocomposites (MAg/VC), which exhibited a great bactericidal and fungicidal potential against *Staphylococcus aureus* ATTC 25923, *Pseudomonas aeruginosa* ATTC 27853, *Escherichia coli* PCM 2209 and *Candida albicans* ATCC 14053. Nanocomposites induced a total eradication of biofilm forming strains in exponential and stationary growth phases. In most cases, the lowest silver content tested (7 mM silver precursor concentration) enabled to eradicate bacteria and fungi in both growth phases. Importantly from the potential biomedical application, beside the pronounced antimicrobial activity, the reduced or totally excluded cytotoxic effect towards A549, HaCaT, and CT26 cell lines was confirmed in the selected *in vitro* model. As the silver nanoparticles become entrapped in the solid polymeric matrix, these bioactive materials could prevent the availability of AgNPs for eukaryotic cells, at the same time preserving the antimicrobial activity in a direct contact with the bacterial surface upon short contact times. The promising result of our research underlines the high application potential of chitosan–ascorbic acid–silver nanocomposites in the biomedical field as *e.g.* wound dressings or medical textiles.

## Conflicts of interest

The authors declare no competing financial interests.

## Supplementary Material

Supplementary information
