# Supplementary material for: Development of noncytotoxic silver–chitosan nanocomposites for efficient control of biofilm forming microbes
Source: RSC Adv. 2017 Nov 13;7(83):52398–413. doi: 10.1039/c7ra08359a (PMC5735359; doi:10.1039/c7ra08359a)
Supplement: Supplementary file 1 [file RA-007-C7RA08359A-s001.pdf]

Electronic Supplementary Material (ESI) for RSC Advances.
This journal is © The Royal Society of Chemistry 2017

Supplementary Information

Development of noncytotoxic silver-chitosan nanocomposites for an efficient control of biofilm forming microbes

Anna Regiel-Futyra[a*], Małgorzata Kus-Liśkiewicz[b], Victor Sebastian[c,d], Silvia Irusta[c,d], Manuel Arruebo[c,d*], Agnieszka Kyzioł[a], Grażyna Stochel[a*]

[a]Faculty of Chemistry, Jagiellonian University, Ingardena 3, 30-060 Kraków, Poland.

[b]Faculty of Biotechnology, Biotechnology Centre for Applied and Fundamental Sciences, University of Rzeszów, Sokołowska 26, Kolbuszowa, 36-100, Poland.

[c]Department of Chemical Engineering and Nanoscience Institute of Aragon (INA), University of Zaragoza, 50018 Zaragoza, Spain.

[d]Networking Research Center on Bioengineering, Biomaterials and Nanomedicine, CIBER-BBN, 50018 Zaragoza, Spain.

[*]Email: regiel@chemia.uj.edu.pl, arruebom@unizar.es, stochel@chemia.uj.edu.pl

Materials and Methods

Metal nanoparticles concentration determination via ICP-MS and ICP-OES measurements

Inductively coupled plasma mass spectrometry (ICP-MS; Elan 6100 Spectrometer) was used for the determination of silver nanoparticles concentration after synthesis. Each sample was diluted in MiliQ water.

Determination of colloidal stability in time

The zeta potential (ζ) of MAg/VC particles were determined *via* dynamic light scattering (DLS; Zetasizer Nano ZS with a 633 nm red laser, Malvern Instruments). Measurements were carried out directly after synthesis and after 60 days to confirm the long-term stability of the obtained colloidal AgNPs.

Fourier transformed infrared spectroscopy (FTIR)

Fourier transformed infrared spectroscopy (FTIR) was used to study the interactions between the polymer functional groups and the metal particles surface. The spectra were collected in the range of 4000-400 cm^{-1} with a 1 cm^{-1} step (Perkin Elmer Spectrum Two ATR).

Contact angle measurements

The wettability of nanocomposites was determined through contact angle measurements *via* the sitting drop technique on a goniometer Surftens Universal (OEG GmbH). Static contact angles of water were calculated using Surftens 4.3-windows image processing software for digital images. High purity water was applied in the measurements with a constant drop volume of 2.5 µL. All measurements were performed at room temperature and the results were presented as mean values (n=10) ± SD.

Cell viability test protocol

Cells after the experiment were washed with PBS, treated with MTT solution (0.5 mg/mL) and incubated for 3 h at 37°C. The formazan crystals were dissolved in a DMSO:CH_3OH mixture (1:1) and the absorbance at 570 nm was measured (Infinite 200 microplate reader, Tecan). A reference wavelength 670 nm was recorded to exclude potential scattering. In the Alamar assay, cells were washed with PBS and incubated with resazurin sodium salt solution (25 µM in PBS) for 3 h at 37°C in the dark. The fluorescence caused by the cellular metabolic activity was measured at 605 nm (excitation wavelength 560 nm).

Results

The mean values of silver concentration were listed in Table S1. In all cases, the obtained concentrations were close but lower than the theoretical values, which might be caused by the dilution error or some losses upon synthesis. The theoretical concentrations were calculated for silver solutions in the polymer solution.

Table S1. The concentration of silver species in chitosan-silver colloids (MAg/VC) after synthesis determined *via* ICP-MS

Sample	Concentration ± SD / mM	Theoretical concentration / mM
**M7/VC**	1.29 ± 0.59	2.00

**M12/VC**	3.63 ± 0.63	4.00
**M26/VC**	6.59 ± 0.46	7.43
**M52/VC**	14.17 ± 3.07	14.86

Colloids stability: Zeta-potential (ζ) of chitosan-silver nanoparticles dispersions

The surface charge of chitosan-silver nanoparticles after synthesis was determined by measurement of the zeta potential at pH = 4.5 (Zetasizer Nano ZS, Malvern Instruments, Malvern, UK). The value of electrokinetic potential determines the colloidal system stability and potential aggregation. The long-term stability was checked two months after synthesis. Samples were stored for this purpose in the dark. Measurements performed in fourfold repetition were presented as mean values ± standard deviation (SD) in Table S2.

Table S2. Zeta potential values for MAg/VCcolloids at pH~4.5.

**Sample**	**ζ ± SD / mV**	
	**1st day**	**60 days**
**M7/VC**	48.5 ± 1.0	45.3 ± 3.5
**M12/VC**	49.7 ± 3.5	46.1 ± 2.9
**M26/VC**	56.1 ± 5.9	52.7 ± 4.4
**M52/VC**	57.8 ± 6.1	55.9 ± 2.8

Zeta-potential values which define stable colloids are generally established for ζ > (±)30 mV.[1] With the increase in the absolute value of the potential, the degree of electrostatic repulsion between nanoparticles increases and thus the more stable the colloidal system is. In such formulations, the aggregation and flocculation are less probable. Chitosan-based silver nanoparticles exhibited positive ζ-potential values, indicating a positively-charged polymeric layer on the AgNPs surface. Values above +45 mV were obtained for all colloids at each silver concentration, which corresponds to a colloidal system with good stability. Depending on the synthetic protocol and applied reducing and stabilizing agent, AgNPs possess positive or negative values of zeta potential *e.g.* negative for sodium borohydride reduced silver nanoparticles or positive for chitosan-based colloids/[2, 3] Since chitosan solution provides

sufficient positive charge coming from the amino groups and also steric stabilization, the obtained silver nanoparticles were electrostatically stable. Moreover, the long-term stability of the colloids was confirmed by almost constant values of zeta potential which is consistent with the literature, where sterically stabilized nanoparticles are considered as more stable than stabilized only by surface charge.[4, 5]

Energy dispersive spectroscopy analysis

The STEM-HAADF micrographs with EDS analysis confirmed the AgNPs chemical identity and presence in the nanocomposites (Figure S1). In all MAg/VC composites with different silver loadings, silver presence was proven; the M26/VC analysis is presented as an example.

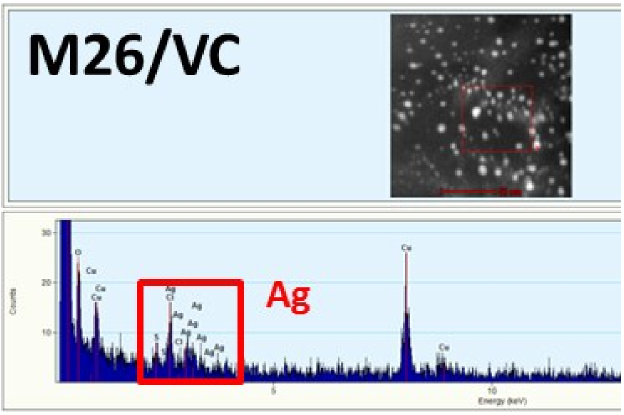

Figure S1. Confirmation of the presence of silver nanoparticles by using Energy Dispersive Elemental Analysis (EDS). The analysis was performed during STEM-HADF mode visualization.

X-Ray photoelectron spectroscopy analysis of MAg/VC composites (XPS)

Table S3 presents the XPS photoelectric peaks positions with atomic % of detected elements and weight percentage calculated for silver. Values of Ag wt.% calculated according to XPS data are close to the theoretical ones with the difference in a 1% range.

Table S3. X-Ray photoelectron spectroscopy analysis of MAg/VC nanocomposites

Sample	Band position and atomic %				Theoretical Ag wt%	XPS Ag wt%
	C 1s	*O 1s*	*N1s*	*Ag 3d*		
**M7/VC**	284.9<br>73.98%	532.9<br>25.27%	399.9<br>4.83%	368.3<br>0.75%	2.9	3.8
**M12/VC**	284.9<br>74.68%	532.6<br>24.25%	399.9<br>3.87%	368.0<br>1.07%	4.9	5.7

**M26/VC**	284.9<br>77.62%	532.8<br>20.40%	389.9<br>4.35%	367.9<br>1.98%	10.1	9.4
**M52/VC**	284.9<br>71.30%	532.6<br>24.89%	389.9<br>5.87%	368.1<br>3.81%	18.3	17.9

Chitosan functional groups – silver nanoparticles surface interactions: IR-ATR measurements

The FTIR spectroscopy was used to confirm the specific interaction of chitosan functional groups with the silver nanoparticles. Spectra of pure chitosan films with medium average molecular weight and MAg/VC composites were collected. A typical chitosan spectrum (Figure S2) presents the characteristic vibrational bands of chitosan at: ~1650 cm^{-1} and ~1590 cm^{-1} corresponding to amide I groups, C-O stretching along with N-H deformation mode (acetylated amine, and to free amine groups, respectively). Bending vibrations of $-CH_2$ and $-CH_3$ may be assigned to the absorption bands at 1376 and 1409 cm^{-1}, respectively.[6] Also, 1320 cm^{-1} and 1259 cm^{-1} bands are detected, corresponding to CH_2 wagging vibration in primary alcohol and the amide III vibration coming from a combination of N-H deformation and C-N stretching.

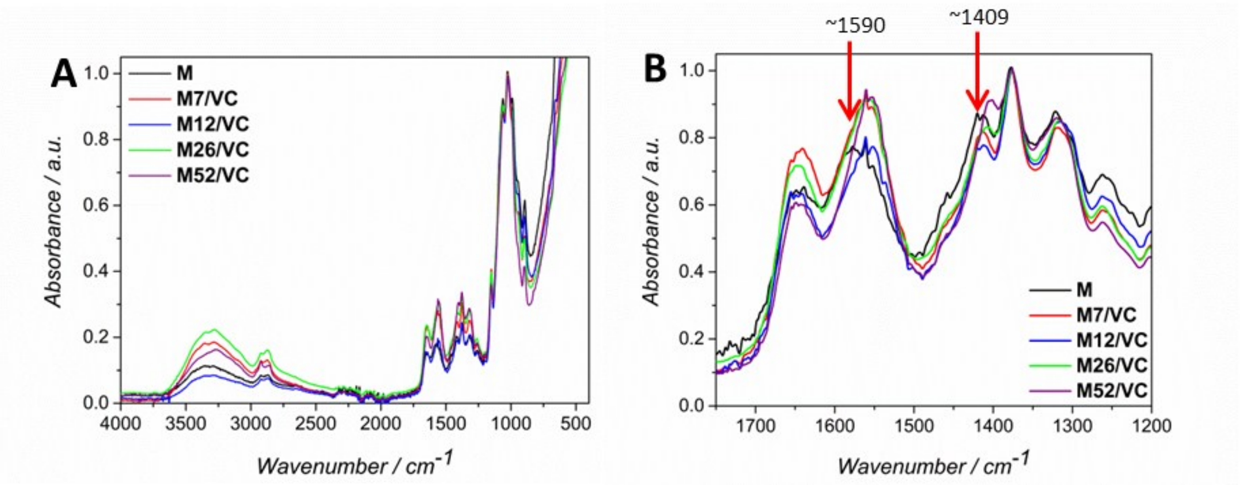

Figure S2. Fourier-Transformed Infrared (FTIR) spectra of pure chitosan medium film and MAg/VC composites with increasing silver NP content in full (A) and narrow wavenumber region (B).

Due to electrostatic interactions between the polymer and the AgNPs, a significant shift to lower wavenumbers for the amino group band (~1590 cm^{-1} for pure polymer) is observed. The second representative shift to lower wavenumbers for chitosan-silver nanocomposites occurs at 1409 cm^{-1} (for pure polymer). The spectra clearly demonstrate the interactions

between the primary amino groups of the polymer with the metal nanoparticles surface.[7, 8] Results stay in agreement with our previous report[9] and results obtained for chitosan-silver materials by Potara *et al.* and Wei *et al.*.[10, 11] Interactions between the polymer and the surface of silver nanoparticles are crucial for the stability of the composites preventing from an excessive release of NPs to the environment.

FTIR analysis was also used to evaluate the potential polymer thermal degradation in the acidic solution used during the synthesis. The spectrum of pure chitosan films with and without applying the temperature conditions, used in the silver nanoparticles synthesis, are presented in Figure S3. The influence of the synthesis protocol on the polymer oxygen-containing groups and potential chitosan degradation were evaluated. In this regard, chitosan medium solution was heated up to 95°C for ~15 h. Afterward, films of chitosan before and after heating were casted and neutralized with1% sodium hydroxide and water.
As described in the main manuscript, the reduction of silver ions in the chitosan solution is coupled with the oxidation and/or hydrolysis of the polymer hydroxyl groups. No significant changes in the shape and position of the band assigned to the hydroxyl groups (~3500cm^{-1}) were observed. The slight intensity increase might be a consequence of the different water content in the films. Based on this finding, it may be assumed that after the temperature treatment, in the absence of silver ions, no oxidation and/or hydrolysis of the polymer hydroxyl groups occur.

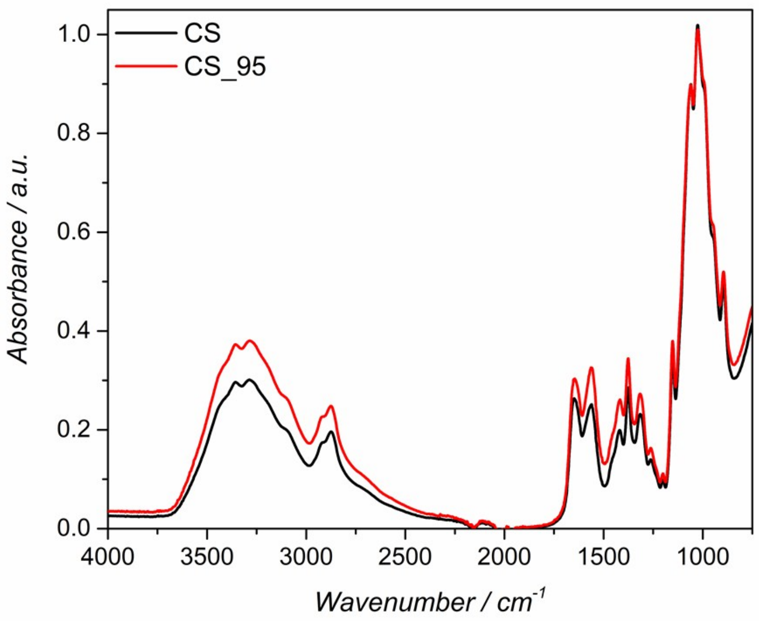

Figure S3. Fourier-Transformed Infrared (FTIR) spectra of chitosan medium film (CS) and chitosan medium film prepared after 95°C/15 h treatment.

Moreover, no significant changes were observed for other bands representing characteristic vibrations of chitosan such as C–O (1250 cm^{-1}), C–O in amide group (~1030 cm^{-1}), or amide I band (~1650 cm^{-1}).

The spectra were also analyzed in the context of potential thermal or acidic degradation of chitosan. Chitosan is typically derived from chitin in the process of alkaline deacetylation at high temperatures ~90-100°C. The deacetylation process leads to the breakdown of amide bonds. Since glycosidic bonds in the chitin chains are very susceptible to acid, typically alkali deacetylation is used[12]. The band present at ~1150 cm^{-1} corresponding to the C–O–C bond in the glycosidic linkage[13], does not exhibit significant changes after the temperature treatment in 0.1 M acetic acid (chitosan solvent). Therefore, it may be concluded that under the applied temperature conditions no glycosidic bonds breakdown occurs. The thermal decomposition of solid chitosan typically occurs at temperatures higher than the one applied in the synthetic procedure here used. Thermogravimetric analysis revealed (data not shown) that the first decomposition stage of chitosan films is located in the range between ~180-350°C. It is a consequence of dehydration of saccharide rings, depolymerization and decomposition of both, acetylated and deacetylation of carbohydrate monomers[14]. The second decomposition stage, thermo-oxidative process, was observed above 500°C[15]. Therefore, 95° is far below the temperature of the polymer degradation.

Next to the thermal, also acidic decomposition of chitosan is possible. Chitosan depolymerization, hydrolysis, fragmentation or even decomposition in hydrochloric acid is well described in the literature. In most of the reports, studies were focused on depolymerization of chitosan using a high concentration of HCl[16-20]. Enzymatic hydrolysis of chitosan is another well-known approach[21]. Acetic acid used for chitosan dissolution is not described as a degradative agent, even at high temperatures.

To conclude, no signs of chitosan depolymerization or degradation was observed on the FTIR spectra after the temperature treatment used.

Contact angle measurements

The most popular approach in the development of antibacterial surfaces is creating hydrophilic or superhydrophobic assemblies since hydrophobic ones with contact angles between 90° and 150° are known to be more favorable for promoting bacterial cells adhesion. However, this phenomenon also strongly depends on cell structure.[22, 23] Another approach is a typical example of antibacterial surface acting by either being toxic when coming into contact

with bacteria or by releasing antibacterial agent from the surface.[24] Nevertheless, the determination of the composites surfaces properties remains as an important aspect of their optimization process and enables for appropriate selection of procedures for nanocomposites *in vitro* biological activity assessment.

The surface properties of CS and CS-Ag films were investigated *via* contact angle measurements. The mean values of contact angles for both, pure chitosan films and nanocomposites containing silver nanoparticles, remain above 90° defining the hydrophilicity/hydrophobicity transition (Table S5).

Table S5. Contact angle values for chitosan and chitosan-silver composites

Contact angle (degrees)	*Chitosan*			
Silver precursor concentration / mM	**L**	**M**	**M/VC**	**H**
**0**	110.0 ± 8.0	107.8 ± 3.8		114.4 ± 3.3
**7**	99.7 ± 1.6	100.7 ± 3.2	113.1 ± 4.0	106.3 ± 7.1
**12**	99.7 ±2.7	110.2 ± 6.7	100.8 ± 2.3	109.3 ± 4.3
**26**	99.5 ± 2.7	100.0 ± 5.0	99.7 ± 3.1	101.5 ± 2.6
**52**	98.0 ± 3.2	98.7 ± 1.3	99.1 ± 5.7	99.8 ± 3.1

The presence of numerous amino groups for chitosan provides a positive charge that promotes wettability, however, neutralization of chitosan films with sodium hydroxide may lead to a depletion of this positive charge and thus the increase in the hydrophobic character. Almeida *et al.*[25] and others reported high contact angles for chitosan films *e.g.* ~95°, attributed to the hydrophobic backbone of chitosan chains.[26] Even though chitosan is known to be rather a hydrophilic polymer, Chen *et al.* demonstrated also quite a high contact angle value ~83.[27] Conversely, chitosan coatings having lower contact angle values (*e.g.* on Ti with contact angle ~76°) and thus a hydrophilic character with good protein and cell adsorption, have been also reported.[28] Interestingly, a dual surface modality of chitosan films was presented by Wang *et al.* who demonstrated the different surface roughness leading to simultaneous hydrophobic and hydrophilic character.[29] For all of our silver-containing films, the hydrophobic character is demonstrated, however, a slight decrease in the contact angle value was observed. The increase of inorganic component as nanofiller in the polymeric matrix may

lead to the increase of the hydrophilic character.[30] This result stays in agreement with Burgers *et al.* who demonstrated anti-adhesive and bactericidal properties of silver-containing dental nanocomposites.[31] Even though a hydrophobic character of MAg/VC composites was proven, the bactericidal and fungicidal effect of silver species together with the bacteriostatic activity of chitosan presumably did not enable for microorganisms attachment and biofilm formation after incubation with the most effective composites as we will demonstrate later on.

Cytotoxicity tests results

The cytotoxicity tests results of chitosan L/M/H based composites containing silver towards three selected cell lines: A549, CT26, and HaCaT are shown in Table S6.

Table S6. The chitosan-silver composites cytotoxicity test results on A549, CT26 and HaCaT cell lines, assessed by Alamar (upper) and MTT (**down, bold**)

		**Cell viability / %**					
		**A549**		**CT26**		**HaCaT**	
**Material**		**S(-)**	**S(+)**	**S(-)**	**S(+)**	**S(-)**	**S(+)**
**LAg**	*L*	107.3 ± 6.8<br>**126.4 ± 19.4**	96.3 ± 1.4<br>**94.6 ± 0.4**	105.5 ± 4.8<br>**111.8 ± 6.8**	96.3 ± 1.4<br>**94.6 ± 1.4**	103.7 ± 10.8<br>**103.3 ± 11.6**	100.6 ± 6.2<br>**100.4 ± 4.8**
	L7	16.1 ± 10.7<br>**19.9 ± 6.4**	99.1 ± 12.6<br>**96.6 ± 4.7**	19.3 ± 14.9<br>**12.5 ± 7.7**	99.1 ± 12.6<br>**96.6 ± 4.7**	5.9 ± 1.4<br>**7.2 ± 0.6**	91.3 ± 14.9<br>**53.9 ± 5.8**
	L12	13.9 ± 9.1<br>**10.2 ± 6.2**	101.9 ± 4.3<br>**94.4 ± 3.1**	21.1 ± 17.4<br>**9.6 ± 5.6**	101.9 ± 4.3<br>**94.4 ± 3.1**	5.3 ± 1.4<br>**7.5 ± 0.6**	85.1 ± 16.5<br>**42.1 ± 5.8**
	L26	6.8 ± 4.1<br>**18.5 ± 14.2**	116.7 ± 10.6<br>**103.9 ± 4.1**	8.8 ± 1.4<br>**6.0 ± 1.3**	116.7 ± 10.6<br>**103.9 ± 4.1**	5.6 ± 1.2<br>**7.0 ± 0.8**	78.4 ± 18.5<br>**11.1 ± 2.5**
	L52	4.5 ± 0.7<br>**4.6 ± 2.4**	92.4 ± 6.33<br>**103.3 ± 5.5**	5.7 ± 1.5<br>**3.9 ± 0.9**	92.4 ± 16.3<br>**103.3 ± 5.5**	4.6 ± 1.5<br>**7.9 ± 0.9**	14.5 ± 8.81<br>**8.2 ± 2.9**
**MAg:1E**	*M*	103.4 ± 2.1<br>**114.9 ± 7.3**	91.9 ± 8.5<br>**109.0 ± 5.2**	102.6 ± 2.1<br>**111.5 ± 4.3**	100.3 ± 3.3<br>**97.5 ± 7.3**	111.4 ± 10.3<br>**95.4 ± 6.4**	97.6 ± 4.1<br>**107.2 ± 11.5**
	M7	3.9 ± 0.1<br>**42.3 ± 10.8**	110.8 ± 2.4<br>**105.5 ± 8.4**	31.7 ± 10.0<br>**16.8 ± 1.9**	101.6 ± 2.2<br>**96.4 ± 5.7**	4.2 ± 1.2<br>**6.6 ± 0.9**	81.9 ± 12.2<br>**85.6 ± 3.6**

	M12	5.3 ± 2.0<br>**3.2 ± 1.3**	87.1 ± 10.1<br>**99.5 ± 3.2**	12.0 ± 6.8<br>**10.1 ± 0.8**	102.6 ± 2.7<br>**90.6 ± 4.3**	4.2 ± 1.2<br>**6.6 ± 2.5**	75.0 ± 15.3<br>**76.4 ± 0.8**
	M26	3.9 ± 0.1<br>**3.5 ± 0.2**	73.1 ± 2.8<br>**108.9 ± 2.3**	7.8 ± 4.4<br>**3.8 ± 1.8**	86.4 ± 13.7<br>**69.1 ± 11.8**	7.8 ± 5.3<br>**7.6 ± 1.0**	60.5 ± 19.5<br>**39.8 ± 29.1**
	M52	3.9 ± 0.1<br>**3.8 ± 0.2**	71.9 ± 3.6<br>**116.5 ± 4.9**	5.9 ± 1.9<br>**3.5 ± 1.3**	65.9 ± 24.0<br>**17.6 ± 6.9**	4.2 ± 1.6<br>**6.6 ± 0.4**	35.5 ± 8.9<br>**25.6 ± 16.2**
**MAg:2E**	*M*	117.7 ± 20.2<br>**107.7 ± 9.3**	100.3 ± 2.9<br>**103.0 ± 2.8**	116.7 ± 21.9<br>**111.5 ± 4.3**	98.9 ± 3.3<br>**96.5 ± 7.7**	98.88 ± 11.9<br>**100.77.5**	98.8 ± 5.7<br>**107.2 ± 3.6**
	M7	96.5 ± 7.5<br>**92.7 ± 29.5**	101.2 ± 8.8<br>**105.8 ± 4.3**	68.9 ± 36.6<br>**116.8 ± 21.1**	102.1 ± 4.8<br>**87.1 ± 21.5**	11.3 ± 1.7<br>**14.2 ± 10.8**	107.8 ± 4.7<br>**107.1 ± 3.6**
	M12	68.9 ± 22.9<br>**51.6 ± 17.6**	104. ± 4.9<br>**102.0 ± 3.8**	36.8 ± 6.3<br>**18.4 ± 2.5**	94.3 ± 3.4<br>**88.4 ± 23.9**	8.9 ± 2.6<br>**9.3 ± 3.8**	99.9 ± 3.1<br>**97.5 ± 4.9**
	M26	5.3 ± 0.7<br>**50.7 ± 10.7**	102.1 ± 2.8<br>**99.9 ± 5.9**	33.1 ± 22.2<br>**11.7 ± 3.3**	97.8 ± 7.7<br>**87.4 ± 4.5**	12.0 ± 5.5<br>**10.8 ± 4.6**	105.4 ± 6.9<br>**94.6 ± 4.2**
	M52	6.4 ± 1.7<br>**37.5 ± 3.6**	101.2 ± 4.0<br>**102.7 ± 3.1**	30.4 ± 24.4<br>**7.0 ± 1.2**	102.4 ± 4.1<br>**80.1 ± 5.3**	8.6 ± 3.6<br>**9.7 ± 4.5**	97.7 ± 5.3<br>**95.8 ± 12.1**
**MAg/VC:1E**	*M*	104.4 ± 1.6<br>**101.0 ± 16.9**	93.2 ± 3.1<br>**99.7 ± 5.2**	105.5 ± 8.4<br>**89.1 ± 2.1**	95.5 ± 1.5<br>**102.6 ± 5.3**	109.7 ± 9.3<br>**111.9 ± 8.2**	97.8 ± 6.0<br>**99.9 ± 1.6**
	M7/VC	7.6 ± 3.1<br>**51.3 ± 11.9**	98.8 ± 9.5<br>**96.9 ± 4.7**	17.5 ± 0.4<br>**33.1 ± 24.7**	95.6 ± 0.4<br>**95.8 ± 2.6**	6.1 ± 1.5<br>**10.5 ± 2.9**	76.8 ± 16.3<br>**85.3 ± 13.1**
	M12/VC	4.3 ± 0.2<br>**4.1 ± 1.3**	100.1 ± 6.1<br>**98.8 ± 1.7**	8.4 ± 5.5<br>**14.3 ± 17.9**	94.2 ± 1.1<br>**40.4 ± 9.3**	5.4 ± 0.5<br>**10.7 ± 2.6**	81.0 ± 14.9<br>**93.3 ± 4.3**
	M26/VC	3.9 ± 0.3<br>**5.1 ± 2.7**	105.4 ± 2.4<br>**103.3 ± 3.5**	6.9 ± 2.6<br>**3.8 ± 2.8**	90.1 ± 11.9<br>**29.7 ± 3.9**	7.9 ± 5.3<br>**10.1 ± 3.6**	74.0 ± 16.9<br>**39.2 ± 4.7**
	M52/VC	4.0 ± 0.5<br>**3.5 ± 1.1**	94.8 ± 17.5<br>**96.6 ± 12.1**	5.1 ± 1.2<br>**4.3 ± 4.5**	43.6 ± 7.5<br>**16.3 ± 2.9**	5.2 ± 0.9<br>**9.8 ± 1.9**	49.1 ± 9.2<br>**13.3 ± 2.2**
**MAg/ VC:2E**	*M*	102.9 ± 7.6<br>**114.4 ± 10.1**	98.6 ± 3.7<br>**96.5 ± 4.1**	102.5 ± 11.8<br>**112.2 ± 8.8**	96.9 ± 4.3<br>**100.5 ± 3.6**	95.1 ± 3.9<br>**102.1 ± 10.2**	101.4 ± 14.2<br>**97.7 ± 4.9**

	M7/VC	82.432.4<br>**46.9 ± 9.4**	104.7 ± 1.0<br>**96.3 ± 5.2**	85.1 ± 23.1<br>**82.6 ± 26.2**	102.2 ± 6.7<br>**97.6 ± 4.3**	49.9 ± 13.1<br>**49.6 ± 10.9**	110.5 ± 11.9<br>**100.7 ± 2.4**
	M12/VC	72.0 ± 32.0<br>**37.3 ± 8.8**	98.3 ± 3.9<br>**97.1 ± 3.7**	50.7 ± 9.2<br>**19.2 ± 11.6**	95.9 ± 4.3<br>**99.3 ± 1.7**	53.3 ± 1.9<br>**30.5 ± 5.5**	101.0 ± 12.4<br>**93.1 ± 8.1**
	M26/VC	57.8 ± 16.9<br>**18.5 ± 12.0**	105.9 ± 2.9<br>**105.4 ± 7.6**	34.5 ± 20.3<br>**12.7 ± 4.7**	104.1 ± 5.8<br>**101.3 ± 4.2**	30.9 ± 10.4<br>**20.9 ± 8.7**	104.2 ± 3.1<br>**98.1 ± 2.6**
	M52/VC	40.0 ± 20.1<br>**13.1 ± 14.5**	101.8 ± 1.6<br>**100.9 ± 3.9**	23.7 ± 14.2<br>**6.9 ± 5.2**	105.0 ± 2.6<br>**101.1 ± 4.0**	6.4 ± 1.1<br>**8.3 ± 3.3**	100.8 ± 6.1<br>**99.3 ± 4.5**
	H	111.9 ± 15.9<br>**95.6 ± 1.9**	101.9 ± 5.2<br>**94.0 ± 2.9**	101.6 ± 3.1<br>**100.4 ± 8.3**	100.0 ± 2.2<br>**100.4 ± 5.6**	99.1 ± 8.5<br>**115.8 ± 23.8**	94.6 ± 2.6<br>**96.5± 1.3**
	H7	4.5 ± 0.4<br>**5.7 ± 6.1**	105.2 ± 7.0<br>**100.5 ± 8.7**	11.8 ± 39.8<br>**8.9 ± 0.1**	110.1 ± 0.4<br>**92.3 ±.6**	6.6 ± 4.4<br>**15.8 ± 14.9**	100.3 ± 9.6<br>**86.4± 0.7**
**HAg**	*H12*	4.4 ± 0.6<br>**5.5 ± 5.9**	101.9 ± 3.6<br>**88.1 ± 7.1**	6.1 ± 2.8<br>**7.9 ± 2.1**	100.9 ± 0.6<br>**90.5 ± 10.9**	5.5 ± 1.2<br>**4.5 ± 1.4**	91.5 ± 1.7<br>**69.5 ± 8.9**
	H26	4.4 ± 0.5<br>**9.5 ± 1.0**	98.9 ± 13.6<br>**92.5 ± 9.3**	6.7 ± 2.3<br>**13.3 ± 13.5**	101.1 ± 4.4<br>**89.1 ± 16.0**	5.0 ± 0.4<br>**4.6 ± 0.9**	86.7 ± 13.6<br>**23.9 ± 27.4**
	H52	5.9 ± 2.1<br>**11.4 ± 0.8**	100.8 ± 4.3<br>**94.4 ± 16.6**	6.0 ± 1.9<br>**7.3 ± 3.8**	76.3 ± 23.2<br>**70.5 ± 12.0**	5.2 ± 1.2<br>**4.5 ± 1.0**	40.8 ± 19.2<br>**15.2 ± 20.0**

References

1. M. Meléndrez, G. Cárdenas and J. Arbiol, *Journal of Colloid and Interface Science*, 2010, **346**, 279-287.
2. S. Agnihotri, S. Mukherji and S. Mukherji, *RSC Advances*, 2014, **4**, 3974-3983.
3. R. Yoksan and S. Chirachanchai, *Materials Science and Engineering: C*, 2010, **30**, 891-897.
4. M. Tejamaya, I. Römer, R. C. Merrifield and J. R. Lead, *Environmental Science & Technology*, 2012, **46**, 7011-7017.
5. C. Lourenco, M. Teixeira, S. Simões and R. Gaspar, *International Journal of Pharmaceutics*, 1996, **138**, 1-12.
6. I. Salcedo, G. Sandri, C. Aguzzi, C. Bonferoni, P. Cerezo, R. Sánchez-Espejo and C. Viseras, *Colloids and Surfaces B: Biointerfaces*, 2014, **117**, 441-448.
7. R. Devi, S. Yadav, R. Nehra, S. Yadav and C. S. Pundir, *J. Food Eng.*, 2013, **115**, 207-214.
8. M. Potara, *Doctoral Thesis Summary*, 2012.

9. A. Regiel, S. Irusta, A. Kyzioł, M. Arruebo and J. Santamaria, *Nanotechnology*, 2013, **24**, 015101.
10. D. Wei and W. Qian, *Colloids and Surfaces B: Biointerfaces*, 2008, **62**, 136-142.
11. E. J. Monica Potara, Annette Damert, Octavian Popescu, Valentin Canpean and Simion Astilean, *Nanotechnology* 2011, **22**.
12. H. K. No and S. P. Meyers, *Journal of Aquatic Food Product Technology*, 1995, **4**, 27-52.
13. F. A. López, A. L. R. Mercê, F. J. Alguacil and A. López-Delgado, *Journal of Thermal Analysis and Calorimetry*, 2008, **91**, 633-639.
14. L. Balau, G. Lisa, M. I. Popa, V. Tura and V. Melnig, *cent.eur.j.chem.*, 2004, **2**, 638-647.
15. G. Lisa, E. Avram, G. Paduraru, M. Irimia, N. Hurduc and N. Aelenei, *Polymer Degradation and Stability*, 2003, **82**, 73-79.
16. K. M. Vårum, M. H. Ottøy and O. Smidsrød, *Carbohydrate Polymers*, 2001, **46**, 89-98.
17. M. R. Kasaai, J. Arul, G. Charlet, #xe9 and rard, *The Scientific World Journal*, 2013, **2013**, 11.
18. O. Scheel and J. Thiem, *Chitin handbook*, 1997, 167-168.
19. A. Einbu and K. M. Vårum, *Biomacromolecules*, 2007, **8**, 309-314.
20. A. Einbu, H. Grasdalen and K. M. Vårum, *Carbohydrate Research*, 2007, **342**, 1055-1062.
21. S.-k. Pan, S.-j. Wu and J.-m. Kim, *Journal of Zhejiang University. Science. B*, 2011, **12**, 931-934.
22. J. Ji and W. Zhang, *Journal of Biomedical Materials Research Part A*, 2009, **88A**, 448-453.
23. G. M. Bruinsma, H. C. van der Mei and H. J. Busscher, *Biomaterials*, 2001, **22**, 3217-3224.
24. E. P. Ivanova, J. Hasan, V. K. Truong, J. Y. Wang, M. Raveggi, C. Fluke and R. J. Crawford, *Applied Microbiology and Biotechnology*, 2011, **91**, 1149-1157.
25. E. V. R. Almeida, E. Frollini, A. Castellan and V. Coma, *Carbohydrate Polymers*, 2010, **80**, 655-664.
26. C.-H. Chen, F.-Y. Wang, C.-F. Mao, W.-T. Liao and C.-D. Hsieh, *International Journal of Biological Macromolecules*, 2008, **43**, 37-42.
27. C.-H. Chen, F.-Y. Wang, C.-F. Mao and C.-H. Yang, *Journal of Applied Polymer Science*, 2007, **105**, 1086-1092.
28. J. D. Bumgardner, R. Wiser, S. H. Elder, R. Jouett, Y. Yang and J. L. Ong, *Journal of Biomaterials Science, Polymer Edition*, 2003, **14**, 1401-1409.
29. X. Wang, Z. Xi, Z. Liu, L. Yang and Y. Cao, *Journal of Nanoscience and Nanotechnology*, 2011, **11**, 9737-9740.
30. B. L. Rao, S. Asha, R. Madhukumar, S. Latha, M. Gowda, G. R. Shetty, K. S. Chandra, P. Naik and Sangappa, *AIP Conference Proceedings*, 2014, **1591**, 807-809.
31. R. Bürgers, A. Eidt, R. Frankenberger, M. Rosentritt, H. Schweikl, G. Handel and S. Hahnel, *Archives of Oral Biology*, 2009, **54**, 595-601.
